# RNA-binding proteins TDP-43 and FUS promote R-loop resolution and regulate transcription termination

**DOI:** 10.1016/j.jbc.2026.111348

**Published:** 2026-03-06

**Authors:** Dorothy Yanling Zhao, Syed Nabeel-Shah, Zuyao Ni, Shuye Pu, Guoqing Zhong, Frank W. Schmitges, Ulrich Braunschweig, Benjamin J. Blencowe, Jack F. Greenblatt

**Affiliations:** 1 The Donnelly Centre, University of Toronto, Toronto, Ontario, Canada; 2 Department of Molecular Genetics, University of Toronto, Toronto, Ontario, Canada

**Keywords:** TDP-43, FUS, SMN, transcription termination, R-loops, ALS, neurodegenerative diseases

## Abstract

TDP-43 and FUS are RNA-binding proteins involved in the regulation of diverse RNA-processing events and have been strongly implicated in neurodegenerative diseases such as amyotrophic lateral sclerosis (ALS) and frontotemporal dementia (FTD). We have previously demonstrated the role of symmetrical dimethylation (me2s) of a conserved arginine residue (R1810 in human POLR2A) in the C-terminal domain (CTD) of RNA polymerase II (RNAPII), which facilitates the recruitment of the Tudor domain-containing protein SMN to resolve R-loops at transcriptional termination sites. Here, we demonstrate that TDP-43 and FUS contribute to transcription termination through the R1810me2s-SMN pathway. Our data show that TDP-43—and to a lesser extent, FUS—are recruited to chromatin *via* this pathway, and that disruption of their recruitment leads to defective RNAPII termination. This impairment results in the accumulation of R-loops and elevated DNA damage to gene terminators. Using transcriptome-wide analyses, we further show that TDP-43 RNA-binding sites are highly correlated with regions of R-loop formation. Importantly, we find that the RNA-binding activity of TDP-43 is essential for its role in resolving R-loops and promoting efficient transcription termination. These findings establish a mechanistic link between TDP-43/FUS, R-loop resolution, and transcription termination, providing new insights into how their dysfunction may drive genome instability and contribute to the pathogenesis of ALS and FTD.

The mammalian POLR2A C-terminal domain (CTD) contains 52 repeats, with the N-terminal half consisting mostly of consensus heptads (Tyr_1_Ser_2_Pro_3_Thr_4_Ser_5_Pro_6_Ser_7_) and the C-terminal half containing many heterogeneous repeats (1). The CTD repeats can be phosphorylated at the residues Tyr1, Thr4, Ser2, Ser5, and Ser7. Ser5 phosphorylation by the general initiation factor TFIIH correlates with transcription initiation and mRNA capping (2, 3). Ser2 phosphorylation by P-TEFb promotes RNAPII promoter release, elongation, and interactions with enzymes required for mRNA 3′-end formation, transcription termination, and histone H3K36 methylation (1, 4, 5). Additionally, the cyclin-dependent kinase CDK12, in complex with cyclin K, phosphorylates Ser2 in the CTD repeats, particularly toward the 3′ ends of long, transcriptionally active genes (6). CDK12 activity is important for maintaining proper expression of genes involved in the DNA damage response, and its loss can impair elongation and polyadenylation site choice (7, 8, 9, 10). Beyond phosphorylation, various other post-translational modifications, such as arginine methylation of the CTD (11), could potentially recruit specialized adaptor proteins to regulate transcription termination and RNA processing.

We have previously shown that a CTD arginine residue (R1810 in human) is symmetrically dimethylated (R1810me2s) and that this R1810me2s modification recruits the adaptor protein SMN (also known as GEMIN1) through the Tudor domain present in SMN (11). SMN self-associates through its N- and C-terminal domains to form a multimeric scaffold for assembling complexes of dimethyl-arginine-containing proteins (12, 13). Because SMN participates in spliceosome assembly and is also present in cytosolic ribonucleoprotein complexes, defects in either function contribute to spinal muscular atrophy (SMA) (12). Complete loss of SMN is embryonic lethal in mammals, and mutations in SMN’s oligomerization or Tudor domain cause the SMA phenotype (14). Senataxin (SETX), a DNA/RNA helicase that is occasionally mutated in ALS (15), is an interaction partner of SMN (16). Both SMN and SETX proteins are involved in transcription termination by RNAPII (11, 17). Mass spectrometry studies have identified over 150 human proteins containing dimethylarginine, and a remarkable number (>50) are involved in RNA metabolism, transcription elongation, and RNAPII termination (Table S1) (18, 19, 20, 21, 22, 23). Therefore, it seems plausible that the CTD R1810me2s-SMN pathway would enhance the association of other Rme2s-containing proteins with the RNAPII CTD for the proper regulation of transcriptional events.

The mutations implicated in amyotrophic lateral sclerosis (ALS) are highly heterogeneous (24) and include an abnormal hexanucleotide expansion in C9orf72, as well as mutations in a variety of unrelated proteins, such as the mitochondrial superoxide dismutase SOD1, the secreted ribonuclease ANG, and several RNA-binding proteins (RBPs), including FUS and TDP-43 (25, 26, 27, 28) that are involved in transcription regulation and alternative splicing (29, 30, 31) (Table S2). The C9orf72 hexanucleotide expansion is found in ∼40% of ALS cases, and the expanded sequences are translated into dipeptide repeats that lead to the formation of inclusion bodies, which trap RBPs such as TDP-43 (32, 33). The pathology caused by SOD1 mutations is also due to the formation of inclusion bodies rather than the lack of dismutase activity (34). FUS and TDP-43 interact with each other, share a number of interaction partners, and are localized in the nucleus and cytoplasm (35, 36, 37). Both FUS and TDP-43 are multi-functional RBPs that play a role in RNA splicing, mRNA transport and stability, and transcription (38, 39). In many types of ALS/FTD, TDP-43 or FUS is known to be trapped in cytoplasmic inclusion bodies (40, 41, 42). Although FUS is mostly nuclear, most FUS mutations that lead to ALS are in its nuclear localization signal sequence and cause it to be trapped in cytoplasmic inclusions (43). TDP-43, also mainly a nuclear protein, is found in cytoplasmic inclusions in almost all cases of ALS (27, 28), although ALS-causing TDP-43 mutations are rare. TDP-43 inclusions coincide with a drastic reduction in normal nuclear TDP-43 localization (44). Therefore, one hypothesis to explain ALS would be that the formation of abnormal cytoplasmic inclusions caused by mutations in many different proteins traps certain RBPs, such as FUS and TDP-43, depleting them from the nucleus where they are needed for gene regulation.

R-loops are three-stranded nucleic acid structures formed during transcription when the RNA hybridizes with the template DNA strand, displacing the non-template strand. R-loops naturally accumulate over G-rich pause sites near transcription termination regions, where their timely resolution is required for efficient release of RNAPII. In particular, SETX, an SMN-interacting DNA/RNA helicase, unwinds R-loops to enable transcription termination by the 5′–3′ exonuclease XRN2 (17). While R-loops serve essential functions in various cellular processes, their abnormal accumulation can interfere with DNA replication, repair, and transcription, ultimately contributing to genomic instability (45, 46, 47, 48, 49, 50). Importantly, increased DNA damage arising from persistent R-loops has been implicated in the neurotoxicity underlying both ALS and FTD (51). Our previous findings indicated that R1810me2s, SMN, and SETX function in an R-loop resolution pathway in transcription termination regions (11). Given SMN’s role as an adaptor for dimethyl-arginine-containing RBPs, we hypothesized that TDP-43 and FUS function downstream of the RNAPII CTD R1810me2s–SMN pathway to resolve R-loops at transcription terminators.

Here we report that among the SMN-interacting proteins, TDP-43 and, to some extent, FUS function downstream of the RNAPII CTD R1810me2s-SMN pathway to resolve R-loops at transcription terminators. Our findings provide mechanistic insight into how defects in transcription termination may underlie neurodegenerative disorders, including SMA, ALS, and FTD, through R-loop accumulation and consequent DNA damage.

## Results

### SMN, TDP-43, and FUS interact with transcription termination factors

Both FUS and TDP-43 are arginine-dimethylated proteins (20, 52) that interact with each other and with SMN (37, 52, 53), and both are implicated in the neurodegenerative disease ALS. Given their established functions in transcription regulation and their ability to bind both DNA and RNA, we asked whether FUS and TDP-43 also function within the RNAPII CTD R1810me2s–SMN pathway. To address this possibility, we first performed immunostaining to assess the subcellular localization of FUS and TDP-43 in HEK293 cells. Consistent with previous reports (54, 55), both proteins predominantly localize to the nucleus, similar to the phosphorylated form of POLR2A and the RNAPII termination factors XRN2 and SETX (Fig. S1). This pattern supports the accuracy of our antibody staining and aligns with their known localization profiles. The observed overlap in immunostaining signals is expected, given the shared nuclear distribution of these proteins (Fig. S1).

**Figure 1:**
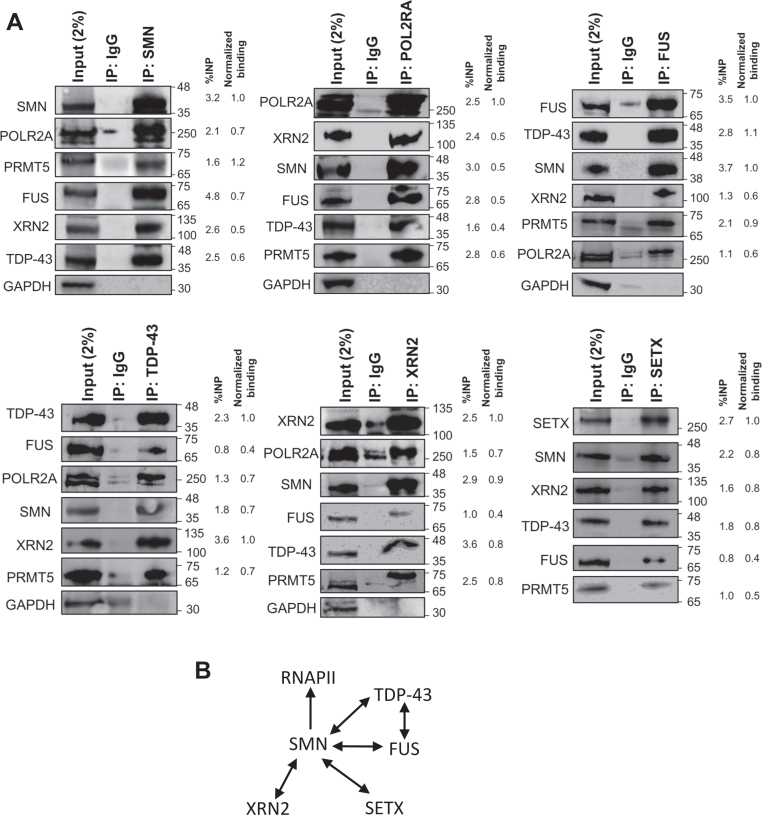
**Interaction network among proteins involved in transcription termination**. *A*, immunoprecipitation (IP) with the indicated antibodies from HEK293 whole cell lysate (WCL), followed by western blotting with the indicated antibodies. IP with IgG serves as a negative control. The blots indicate that POLR2A, SMN, FUS, TDP-43, PRMT5 and the termination factors SETX and XRN2 interact directly or indirectly with each other. Each co-IP was performed with multiple biological replicates (*n = 3–4*). Quantifications of band intensities are indicated on the right side of each panel for the experimental IP lanes, with signals from the IgG lanes subtracted. *B*, a summary of the interactions detected by co-IP experiments. All of these interactions, except the SMN-XRN2 and SMN-TDP-43 interactions, have previously been reported (11, 16, 106). Bands observed in the IgG lanes in some blots are due to spillover from adjacent lanes. The slight curvature of the TDP-43 band in the XRN2-IP lane is attributable to a localized air bubble that occurred during transfer. Since IP materials were split and resolved in parallel to probe for proteins with similar molecular weights, we quantified bands using both %INP and normalized binding (IP: prey/bait) methods for consistency (see methods). All IPs were performed using cell lysates treated with Benzonase. Membranes were often cut based on known molecular weights of proteins prior to antibody probing.

We next performed co-immunoprecipitation (co-IP) experiments using SMN as a bait and observed that both FUS and TDP-43 interact, directly or indirectly, with SMN (Fig. 1*A*). RNAPII (POLR2A) and termination factors such as SETX and XRN2 also interact with SMN, consistent with prior findings (11). Reciprocal co-IPs further confirmed robust interactions of TDP-43 or FUS with SMN, RNAPII (POLR2A), PRMT5, SETX, and XRN2, in addition to the known interaction between FUS and TDP-43 (Fig. 1*A*). These findings are summarized in Figure 1*B*. Importantly, all lysates were treated with a promiscuous nuclease, Benzonase, prior to IPs to minimize DNA/RNA-mediated interactions. Many of these protein-protein interactions persist even after a 3-days α-amanitin treatment that eliminates the bulk of RNAPII in HEK293 cells (Fig. S2*A*), indicating that they occur independently of RNAPII. Given that FUS, TDP-43, XRN2, and SETX all interact with PRMT5 and contain dimethylated arginine (Table S1) (18, 19, 20, 21, 22, 23), these proteins are likely direct PRMT5 substrates.

### Recruitment of TDP-43 to RNAPII is mediated by SMN and CTD R1810

**Figure 2:**
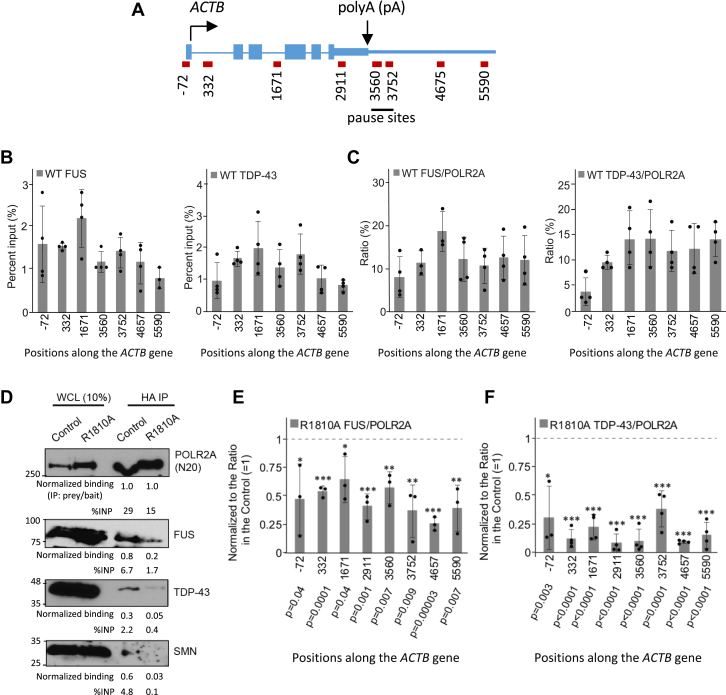
**R1810 on the RNAPII CTD enhances the recruitment of SMN, FUS, and TDP-43**. *A*, primer positions along the *ACTB* gene; the termination pause sites are underlined. *B* and *C*, quantification of ChIP signals in HEK293 cells, expressed as percent input (%INP) (*B*), or as ratios (*C*) of FUS and TDP-43 %INP to RNAPII %INP values at the indicated *ACTB* gene regions. Error bars denote standard deviation (s.d.) of biological replicates (*n = 3–4*). %INP in panel *B* reflects absolute ChIP enrichment, whereas the ratios in *panelC* normalize FUS or TDP-43 recruitment to the amount of RNAPII present at the same genomic position, thereby reporting the recruitment efficiency of FUS or TDP-43 to RNAPII. *D*, IP with the indicated antibodies from WCL from Raji cells stably expressing α-amanitin-resistant HA-tagged wild-type or R1810 A mutant POLR2A after α-amanitin treatment that abolishes the endogenous POLR2A. HA-tagged wild-type (Control) or R1810 A mutant POLR2A was precipitated with anti-HA and analyzed by western blotting with the indicated antibodies (*n = 2*; also see Fig. S2*B*). Values from the quantification of bands using both %INP and normalized binding (prey/bait) methods are shown below each panel. *E* and *F*, quantification of ChIP signals in Raji cells as ratios of FUS %INP to POLR2A %INP (*E*) or TDP-43 %INP to POLR2A %INP (*F*) showing the relative effects of the POLR2A R1810 A mutation. Ratios in the wild type R1810 controls were normalized to one (indicated as dotted lines). Error bars denote s.d. of biological replicates (*n = 3–4*). All qPCR *p*-values were calculated using two-tailed Student’s t-tests comparing mutant to control groups, where ∗∗∗ indicates *p* ≤ 0.001, ∗∗*p* ≤ 0.01, and ∗*p* ≤ 0.05.

The protein–protein interaction data described earlier suggested that TDP-43 and FUS might participate in our previously identified R-loop resolution pathway involving R1810me2s, SMN, and SETX (11). To explore this possibility, we initially performed chromatin immunoprecipitation (ChIP) followed by quantitative PCR (qPCR) for FUS and TDP-43, utilizing the *ACTB* as a model gene locus in these experiments (Fig. 2*A*). In Figure 2*B*, we present the ChIP-qPCR signals for FUS and TDP-43 expressed as percent input (%INP), which reflects the absolute enrichment of each protein at the indicated *ACTB* regions. In Figure 2*C*, we normalized the FUS and TDP-43 ChIP signals to the corresponding RNAPII %INP values at each site. This ratio represents the amount of FUS or TDP-43 that is recruited per unit of RNAPII and, therefore, reflects recruitment efficiency rather than absolute occupancy. Normalizing to RNAPII is essential, because RNAPII levels can vary across conditions or genomic positions, and the ratio allows us to determine the efficiency of the recruitment of particular proteins to RNAPII. These experiments showed that both FUS and TDP-43 associate with the *ACTB* gene throughout its length from the promoter to the termination regions (Fig. 2, *B* and *C*).

We previously showed that the RNAPII CTD R1810me2s modification enhances SMN-mediated recruitment of SETX to RNAPII (11). To determine whether TDP-43 and FUS recruitment to RNAPII is similarly dependent on RNAPII CTD R1810, we performed co-IP experiments using HA-tagged POLR2A constructs, either the wild type or the R1810A mutant. Interactions of TDP-43 or FUS with HA-POLR2A decrease when R1810 is mutated to alanine (A) on the POLR2A CTD (Fig. 2*D*, Fig. S2*B*), with a relatively more drastic effect for TDP-43. These experiments were performed in Raji cells after 3 days of α-amanitin treatment (2 μg/ml) that removes the bulk of the endogenous RNAPII (Fig. S2*A*). To further characterize the role of CTD R1810me2s in recruiting TDP-43 or FUS to RNAPII associated with chromatin, we performed ChIP assays in Raji cells, expressing α-amanitin-resistant, wild-type or R1810A POLR2A, after 3 days of α-amanitin treatment. ChIP-qPCR signals for FUS/POLR2A and TDP-43/POLR2A decrease across the *ACTB* gene in the RNAPII R1810 A mutant, with a more pronounced reduction for TDP-43 (Fig. 2, *E* and *F*).

**Figure 3:**
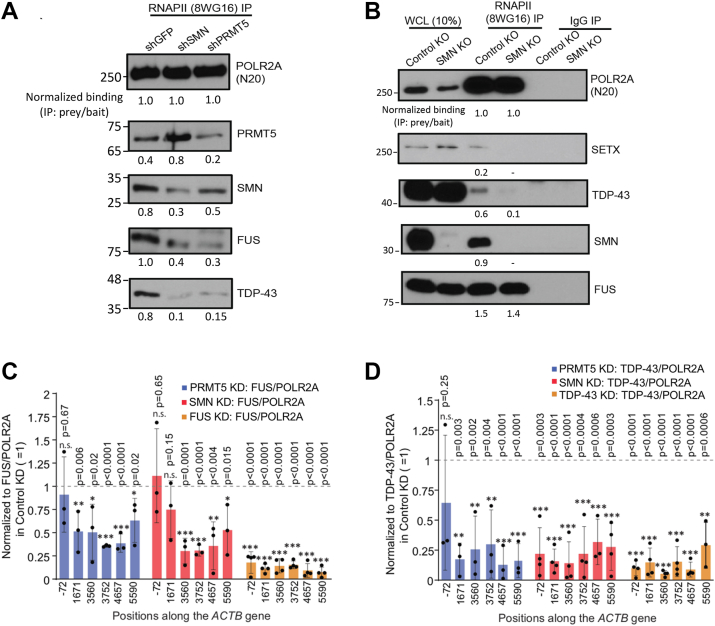
**The loss of PRMT5 or SMN disrupts the interactions among RNAPII, SMN, FUS, and TDP-43**. *A*, IP with 8WG16 antibodies for POLR2A from HEK293 WCL upon stably knocking down PRMT5 or SMN, with the knockdown of GFP as negative control. Western blots were performed with the indicated antibodies. The knockdown of PRMT5 or SMN causes a reduction of TDP-43 and FUS (to some extent) interaction with RNAPII. Knockdown IP blots were performed alongside our previously reported data (extended data Fig. 1h in Zhao *et al*. (11)). All co-IP experiments were performed in three biological replicates (PRMT5 knockdown (TDP-43 and FUS), SMN knockdown (TDP-43 and FUS), *n = 3*). *B*, IP with the indicated antibodies from HEK293 WCL, upon knocking out of SMN with the CRISPR/Cas9 system or using scrambled guide RNA as negative control. Western blots were performed with the indicated antibodies to show that the SMN knockout leads to a loss of interaction of TDP-43 and SETX with POLR2A (*n = 3*). IP with IgG serves as a negative control. For both *A* and *B*, bands were quantified, and normalized binding (IP: prey/bait) values are shown below each panel. *C* and *D*, Quantification of ChIP-qPCR signals shown as ratios of FUS ChIP % input to POLR2A ChIP % input (*C*) or TDP-43 ChIP % input to POLR2A ChIP % input (*D*). For each genomic position, the ratio in control (*i*.*e*., knockdown against GFP) was normalized to 1 (*dotted line*) and used as the reference for statistical comparison. Error bars represent s.d. of biological replicates (*n = 3–4*). *p*-values were calculated using two-tailed Student’s t-tests comparing each knockdown condition to its corresponding control at the same genomic position (control set to 1): ∗∗∗*p* ≤ 0.001, ∗∗*p* ≤ 0.01, ∗*p* ≤ 0.05, n.s., not significant.

Additionally, by immunoprecipitating RNAPII, we found that depletion of SMN or PRMT5 using shRNAs reduces the association between RNAPII and TDP-43, as reflected by the quantified co-IP signals for RNAPII and TDP-43 (Fig. 3*A*). In contrast, the interaction between RNAPII and FUS shows only a modest reduction upon SMN depletion, consistent with the ability of FUS to bind directly to the RNAPII CTD (29, 56, 57). Likewise, knockout of SMN using CRISPR/Cas9-mediated mutagenesis in HEK293 cells strongly diminishes the RNAPII–TDP-43 and RNAPII–SETX interactions but has a less pronounced effect on the RNAPII–FUS interaction, as the signal remains readily detectable (Fig. 3*B*). Of note, TDP-43 and FUS protein levels remain unaltered in SMN and PRMT5 depletion cells in comparison with controls (Fig. S2*C*). Because PRMT5 regulates multiple methylation events, these experiments report the effect of PRMT5 depletion rather than directly measuring loss of CTD R1810me2s. Nonetheless, these results are consistent with our previously published biochemical evidence that PRMT5 symmetrically dimethylates R1810 on the RNAPII CTD (11). Next, using ChIP assays, we found that PRMT5 or SMN knockdown reduces the association of TDP-43 with POLR2A across the *ACTB* gene body, with the strongest effects at the pause/termination site (*i*.*e*., position 3560) (Fig. 3, *C* and *D*). In contrast, the effect on FUS is modest and most evident at the pause sites, consistent with the ability of FUS to bind RNAPII directly. These ChIP experiments also confirmed the specificity of the FUS and TDP-43 antibodies. Together with the co-IP analyses, these findings indicated that SMN and PRMT5 enhance the association of TDP-43 and, to a lesser extent, FUS with transcribing RNAPII.

### Endogenous RNAPII R1810A mutant is defective in RNAPII termination

**Figure 4:**
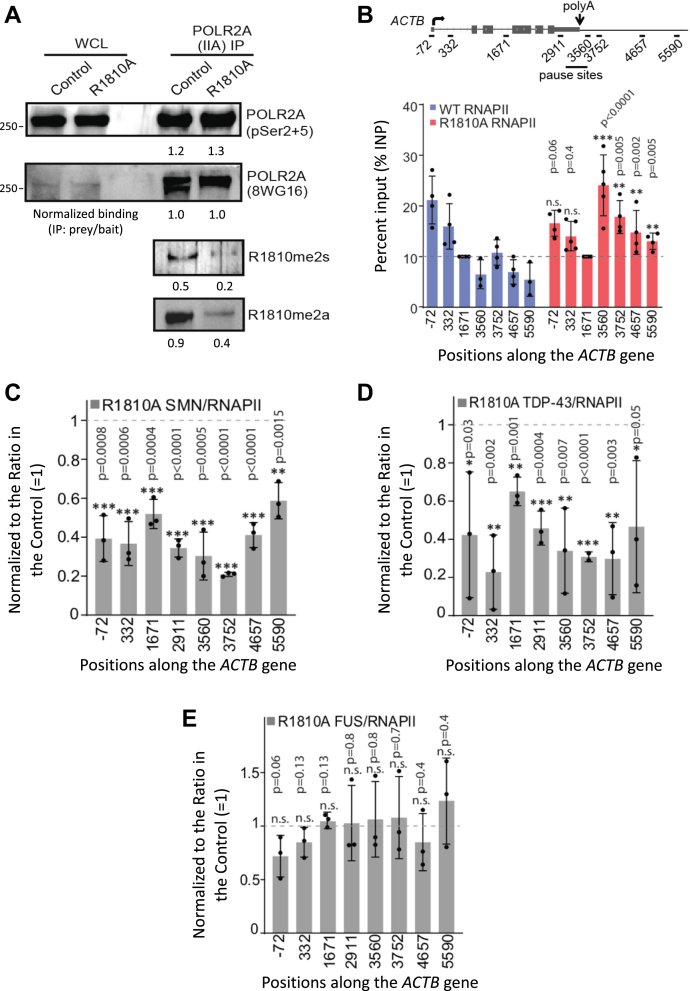
**Stable endogenous RNAPII CTD R1810 A mutant exhibits RNAPII termination defects and reduced SMN and TDP-43 recruitment**. *A*, immunoprecipitation (IP) with the indicated antibodies from HEK293 whole cell lysate (WCL), followed by western blotting with the indicated antibodies. The blots indicate that the R1810 A mutation on RNAPII causes the loss of both R1810me2a and R1810me2s signals as detected by specific antibodies. Bands were quantified and normalized binding (IP: prey/bait) values are shown below each panel. *B*, quantification of RNAPII ChIP for WT or the R1810 A mutant POLR2A with antibodies (8WG16, N20) in HEK293 cells, using the indicated primer positions for qPCR along the *ACTB* gene. ChIP signals were normalized to the gene body region (1671; *dotted line*) across samples and replicates. Error bars denote s.d. of biological replicates (*n = 3–5*). *p*-values were calculated using two-tailed Student’s *t* test for the indicated sites, ∗∗∗*p* ≤ 0.001, ∗∗*p* ≤ 0.01, ∗*p* ≤ 0.05, n.s., non-significant. *C*, *D*, and *E*. quantification of ChIP data in HEK293 cells as SMN/POLR2A (*C*), TDP-43/POLR2A (*D*), or FUS/POLR2A (*E*) ratio to show the relative effects of mutating R1810 to alanine, with the signals in the wild type R1810 control normalized to 1 (*dotted line*). Error bars denote the s.d. of biological replicate (*n = 3*). *p*-values were calculated using two-tailed Student’s *t* test against normalized control that is set to 1, where ∗∗∗*p* ≤ 0.001, ∗∗*p* ≤ 0.01, ∗*p* ≤ 0.05, n.s., non-significant.

In our previous studies (11) and in Figure 2, we used α-amanitin to eliminate endogenous RNAPII and assay the transient effect of the R1810A mutation in α-amanitin-resistant HA-tagged POLR2A. To examine the stable effect of this mutation, we generated CRISPR/Cas9-mediated knock-in of the mutation in the endogenous POLR2A gene in HEK293 cells. After confirming through DNA sequencing that R1810 on both alleles of the endogenous POLR2A were mutated to alanine, we then precipitated wild-type and R1810A mutant RNAPII using antibodies against the hypophosphorylated IIA (8WG16) form of the CTD. As expected, the R1810A mutant RNAPII shows a loss of signal for both R1810me2a and R1810me2s modifications (Fig. 4*A*), as detected by their specific antibodies (11, 58), confirming that R1810 on the endogenous POLR2A was successfully mutated. We next performed RNAPII ChIP-qPCR and observed that the R1810A mutant accumulates at higher levels in the termination regions of the *ACTB* and the *GAPDH* genes (Fig. 4*B*, Fig. S3*A*), in agreement with our results using α-amanitin-resistant HA-POLR2A in Raji cells (11).

ChIP-qPCR along the *ACTB* gene shows reduced SMN/RNAPII and TDP-43/RNAPII ratios in the endogenous RNAPII R1810 A mutant compared with wild-type RNAPII (normalized to 1), indicating impaired recruitment of these factors (Fig. 4, *C* and *D*), similar to what we observe with α-amanitin-resistant HA-tagged POLR2A (Fig. 2*F*) (11). However, FUS/RNAPII ChIP signals show little or no reduction, likely reflecting alternative or compensatory modes of RNAPII association that do not depend on R1810 of the CTD (Fig. 4*E*) or SMN (Fig. 3*B*).

### FUS and TDP-43 are important for transcription termination by RNAPII

**Figure 5:**
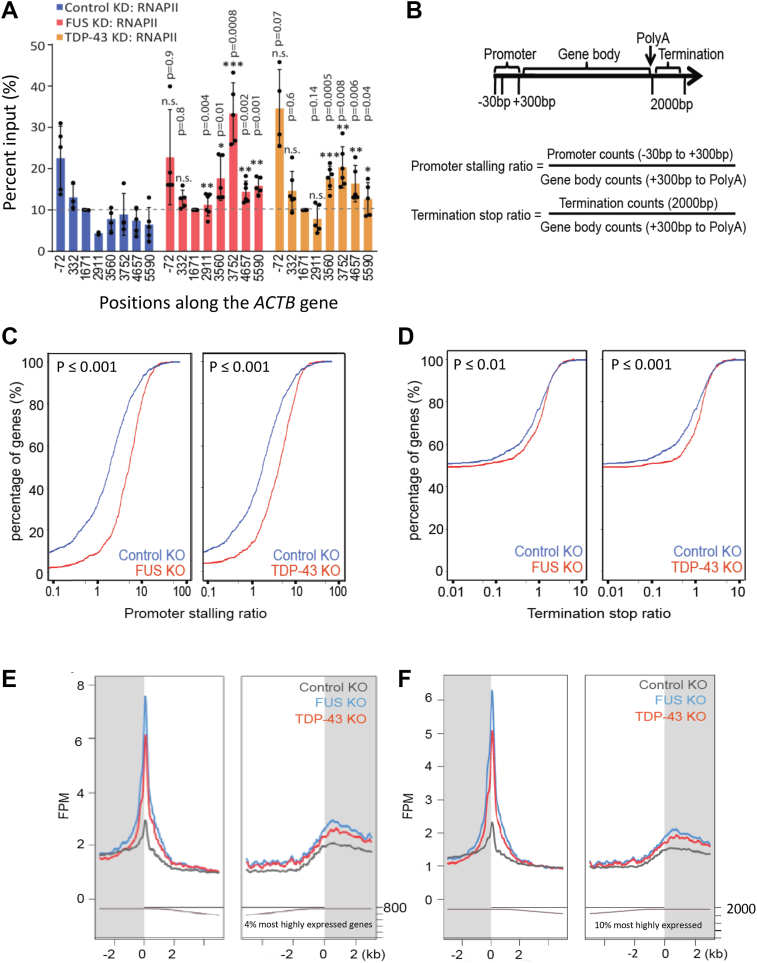
**FUS and TDP-43 regulate genome-wide transcription termination by RNAPII**. *A*, quantification of RNAPII ChIP using POLR2A antibody (4H8, N20) in HEK293 cells, using the indicated primer positions for qPCR along the *ACTB* gene after stably knocking down FUS or TDP-43, with GFP knockdown as a negative control. ChIP signals are normalized to the gene body region (1671; dotted line) across samples and replicates. Error bars denote s.d. of biological replicates (*n = 3–5*). *p*-values were calculated using two-tailed Student’s *t* test for the indicated pause sites, ∗∗∗*p* ≤ 0.001, ∗∗*p* ≤ 0.01, ∗*p* ≤ 0.05, n.s.: non-significant. *B*, schematics for the calculation of the stalling ratio at the promoter region and stop ratio at the termination region. A rightward shift of the curve in the plots indicates increased RNAPII accumulation in the corresponding regions. *C* and *D*, RNAPII ChIP-seq (with the N20 antibody in HEK293 cells). Compared to the scrambled KO control, FUS or TDP-43 KO leads to increased RNAPII accumulation in the promoter (*C*) and termination (*D*) regions of many genes. Top 10% genes (∼2000 genes) by expression were analyzed. Statistically significant differences between the quantiles were determined using the Kolmogorov Smirnov test; level of significance is set at *p* ≤ 0.05. *E* and *F*, a second way of displaying ChIP-seq analysis in which the reads are normalized to the gene body. The knock-out of FUS or TDP-43 leads to increased RNAPII accumulation in the promoter and termination regions of many genes. Top 800 (*E*) or 2000 (*F*) genes by expression with reliable ChIP-seq signals were analyzed; the number of genes analyzed is displayed on the *bottom right* of the graph.

Because SETX, SMN, and the RNAPII CTD R1810me2s modification are important for transcription termination (11, 17), we investigated whether the loss of FUS or TDP-43 produces similar defects. Upon stable shRNA-mediated knockdown or CRISPR/Cas9-mediated knockout of FUS or TDP-43 in HEK293 cells (Fig. S3*B*), RNAPII accumulates in the termination regions of the *ACTB* gene (Fig. 5*A*, Fig. S4) and *GAPDH* gene (Fig. S3, *C*−*D*).

We next performed RNAPII ChIP-seq to determine whether the loss of FUS or TDP-43, like the loss of SMN or the presence of an R1810 A mutation in the RNAPII CTD, broadly affects transcription termination. Using HEK293 cells with CRISPR/Cas9-mediated knockout of FUS or TDP-43, or a scrambled guide RNA control, we generated RNAPII ChIP-seq datasets (with the N20 antibody), with 12 to 20 million unique reads per sample. For the analysis, we focused on genes with robust ChIP-seq signals (top four or 10% by expression), as sequencing depth and background interference become issues when more genes are included. Cumulative distribution plots were generated for the promoter-stalling ratio and the terminator stop ratio, calculated as promoter or terminator region reads normalized to gene body reads, respectively (Fig. 5, *B*−*D*). A rightward shift of the plot reflects increased RNAPII accumulation in the corresponding regions (Fig. 5, *B*−*D*). Loss of FUS or TDP-43 produces substantial genome-wide accumulation of RNAPII in promoter regions, in agreement with the literature for FUS depletion (Fig. 5, *C* and *D*, *F*) (29, 56). Supporting the roles of FUS and TDP-43 in the regulation of RNAPII termination, there is widespread accumulation of RNAPII in the termination regions when either protein is depleted (Fig. 5, *D*−*F*; Fig. S5).

### TDP-43 regulates RNAPII transcription termination *via* R-loop binding

**Figure 6:**
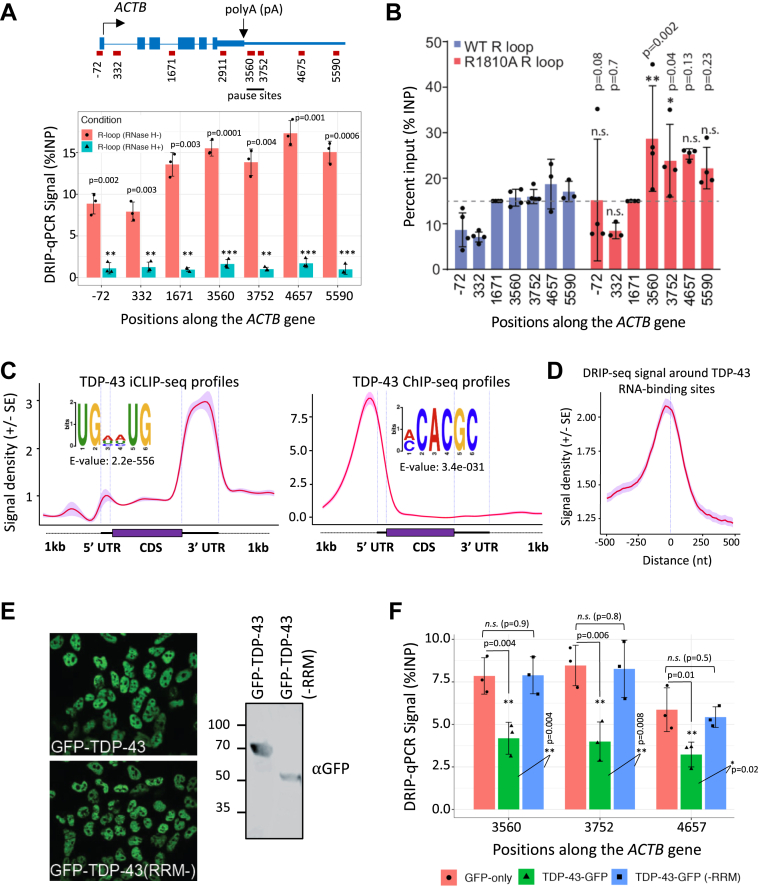
**TDP-43 regulates transcription termination *via* R-loop binding**. *A*, quantification of R-loops with the S9.6 antibody in HEK293 cells with or without RNase H treatment on the *ACTB* gene. Error bars denote s.d. of biological replicates (*n = 3*). *p*-values were calculated using two-tailed Student’s *t* test, ∗∗∗*p* ≤ 0.001, ∗∗*p* ≤ 0.01, ∗*p* ≤ 0.05, n.s., non-significant. *B*, quantification of R-loops with the S9.6 antibody in HEK293 cells for the WT or the R1810 A mutant POLR2A, using the indicated primer positions for qPCR along the *ACTB* gene. Signals are normalized to the gene body region (1671; *dotted line*) across samples and replicates. Error bars denote s.d. of biological replicates (*n = 3–4*). *p*-values were calculated using two-tailed Student’s *t* test for the indicated sites, ∗∗∗*p* ≤ 0.001, ∗∗*p* ≤ 0.01, ∗*p* ≤ 0.05, n.s.: non-significant. *C*, standardized metaplot profiles showing the normalized RNA- and DNA-binding densities of TDP-43 using iCLIP-seq (*left*) and ChIP-seq (*right*) datasets, respectively. Shaded area represents standard error (SE). Enriched sequence motifs found in TDP-43 iCLIP-seq and ChIP-seq peaks are also depicted. *D*, Average R-loop density, as estimated using the DRIP-seq data, around the iCLIP-seq identified TDP-43 RNA-binding sites. *E*, *left*, GFP immunofluorescence verifies expression in the nucleus of cells expressing GFP-TDP-43 and truncated GFP-TDP-43 (RRM-) after 1 day of induction with doxycycline. Note that the full-length GFP-TDP-43 Immunofluorescence image is the same as that shown in Figure S1*D*. *Right*, Western blotting with GFP antibody to show the expression levels and sizes of the GFP-TDP-43 fusion proteins in the HEK293 whole cell lysates. The truncated TDP-43 lacks RRMs at the *C* terminus of the protein. *F*, quantification of R-loops in the *ACTB* gene upon doxycycline-mediated induction of the indicated transgene. Error bars denote s.d. of biological replicates (*n = 3*). *p*-values were calculated using two-tailed Student’s *t* test, ∗∗*p* ≤ 0.01, ∗*p* ≤ 0.05, n.s., non-significant.

We and others previously established that efficient resolution of R-loops that accumulate in transcription termination regions depends on the RNAPII CTD R1810me2s–SMN–SETX pathway (11, 17). The monoclonal antibody S9.6 specifically recognizes RNA/DNA hybrids (R-loops), as demonstrated by loss of signal after RNase H treatment (Fig. 6*A*) (11). Consistently, we find that the endogenous RNAPII R1810 A mutation leads to elevated R-loop accumulation within the termination regions of the *ACTB* (Fig. 6*B*) and *GAPDH* (Fig. S6*A*) genes. These regions correlate closely with sites of RNAPII stalling, reinforcing the link between R-loop persistence and impaired termination in the R1810A mutant.

Given that both FUS and TDP-43 are RNA-binding proteins with the potential to influence co-transcriptional RNA processing, we asked whether their RNA-binding activities may limit R-loop formation by shielding nascent RNA from hybridizing to template DNA. We focused on TDP-43 because its ChIP signal is markedly reduced in R1810 A mutants, in a manner comparable to that of SMN (Fig. 4, *C* and *D*). By integrating publicly available TDP-43 ChIP-seq datasets with our recently reported iCLIP-seq data in HEK293 cells (59, 60), we observed that TDP-43 exhibits distinct DNA- and RNA-binding profiles (Fig. 6*C*). Supporting the idea that RNA-binding by TDP-43 influences R-loop formation in transcription termination regions, the iCLIP-seq profile revealed that TDP-43 is strongly enriched at 3′ UTRs of target transcripts (Fig. 6*C*). Peak distribution analysis indicated that the majority of the ChIP peaks reside within promoter regions, whereas the iCLIP-seq peaks fall in the introns and 3′UTRs (Fig. S7, *A*−*B*). Motif analysis further supported this distinction: UG-rich motifs are enriched in iCLIP-seq peaks, whereas C-rich motifs are found in the ChIP-seq peaks (Fig. 6*C*).

To evaluate whether TDP-43 RNA-binding sites coincide with zones of R-loop formation in the termination regions, we analyzed published DNA/RNA-IP-seq (DRIP-seq) datasets from HEK293 cells (61). We found that R-loops exhibit strong enrichment around the RNA-binding sites of TDP-43 (Fig. 6*D*), supporting a role of RNA-binding by TDP-43 in R-loop metabolism. This is consistent with observations that the loss of RNA-binding activity in TDP-43 mutants disrupts their ability to suppress R-loops (62). TDP-43 contains two RNA recognition motifs (RRMs) that are essential for its RNA-binding activity. To directly test the requirement of TDP-43 RNA-binding in R-loop resolution, we generated HEK293 Flp-In T-REx cell lines that stably express either GFP-tagged wild-type TDP-43 or a truncation mutant lacking both RRMs (Fig. 6*E*). Notably, deletion of both RRMs does not alter TDP-43’s nuclear localization (Fig. 6*E*, left). Using these cell lines, we then performed RNAPII ChIP-qPCR and DRIP-qPCR assays to evaluate the role of TDP-43 RNA binding in transcription termination and R-loop resolution, respectively. With RNAPII ChIP signals normalized in gene bodies to a control that overexpresses wild-type TDP-43, we found that the overexpression of the truncation mutant indeed increases accumulation of RNAPII in the termination regions of the *ACTB* and *GAPDH* (Fig. S8, *A*−*B*) genes. Moreover, overexpression of wild-type TDP-43 significantly reduces R-loop accumulation at the examined *ACTB* and *GAPDH* loci relative to GFP-only controls, whereas the truncation mutant fails to do so (Fig. 6*F*; Fig. S8*C*). These findings indicated that the RNA-binding ability of TDP-43 is essential for its role in RNAPII termination and R-loop resolution, perhaps through sequestering the nascent RNA and preventing its hybridization to the single-stranded DNA template behind the transcription bubble.

### FUS and TDP-43 prevent the accumulation of R-loops and DNA damage at termination sites

R-loop accumulation during transcription is associated with DNA damage and genome instability (46, 63, 64, 65). We therefore examined this relationship in the context of FUS or TDP-43 loss.

**Figure 7:**
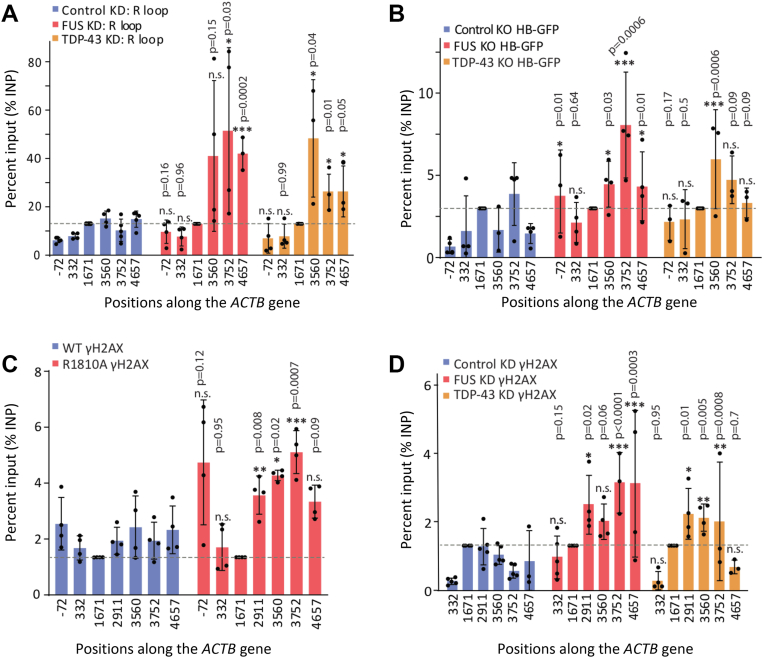
**FUS and TDP-43 prevent accumulation of R-loops and DNA damage at RNAPII transcription terminators**. *A*, Quantification of R-loops with the S9.6 antibody in HEK293 cells with or without knockdown of FUS or TDP-43, using the indicated primer positions for qPCR along the *ACTB* gene. R-loop signals are normalized to the gene body region (1671; *dotted line*) across samples and replicates. Error bars denote s.d. of biological replicates (*n = 3–5*). *B*, ChIP quantification of R-loops with the GFP-HB construct, with the indicated primer positions for qPCR along the *ACTB* gene, after knocking out FUS or TDP-43 by CRISPR/Cas9 mutagenesis, using scrambled guide RNA as a negative control. Error bars denote s.d. of biological replicates (*n = 3–4*). *C* and *D*, ChIP quantification of γH2AX as percent input in HEK293 cells, along the length of the *ACTB* gene, between WT and the endogenous R1810 A mutant POLR2A (*C*), or after knocking down FUS or TDP-43, with GFP knockdown as control (*D*). Error bars denote s.d. of biological replicates (*n = 3–5*). R-loop and ChIP signals are normalized to the gene body region (1671; *dotted line*) across samples and replicates. For all qPCR experiments, *p*-values were calculated using two-tailed Student’s *t* test for the indicated sites, ∗∗∗*p* ≤ 0.001, ∗∗*p* ≤ 0.01, ∗*p* ≤ 0.05, n.s., non-significant.

Using the S9.6 antibody to detect R-loops, we found that knockdown of FUS or TDP-43 leads to increased R-loop accumulation within the termination regions of the ACTB gene (Fig. 7*A*) where RNAPII stalls, similar to the effects observed with the RNAPII R1810 A mutation or the loss of SMN or SETX (11). We further validated R-loop detection with an alternative strategy by using a stably expressed GFP fusion protein containing the RNase H1 R-loop-binding domain (GFP-HB) (66). Consistently, anti-GFP ChIP for GFP-HB detects increased R-loop accumulation in the termination regions of *ACTB* (Fig. 7*B*) and *GAPDH* (Fig. S6*B*) when FUS or TDP-43 is knocked out. These results indicated that RNAPII CTD R1810me2s, SMN, SETX, FUS, and TDP-43 collectively contribute to R-loop resolution and proper transcription termination by RNAPII.

We next examined DNA damage at transcription terminator regions upon the loss of FUS or TDP-43 by performing γH2AX ChIP as a proxy for the accumulation of DNA damage (67) (Fig. 7, *C* and *D*; Fig. S9, *A*−*B*). Loss of FUS or TDP-43 indeed leads to increased γH2AX signal and elevated γH2AX/H2AX ratios at the *ACTB* termination regions, consistent with increased RNAPII stalling and R-loop accumulation. Similar increases occur when R1810 is mutated to alanine in the endogenous POLR2A gene. Together, these findings support the idea that FUS and TDP-43, along with RNAPII CTD R1810me2s, SMN, and SETX, function to suppress R-loop accumulation and prevent DNA damage at transcription termination sites.

## Discussion

**Figure 8:**
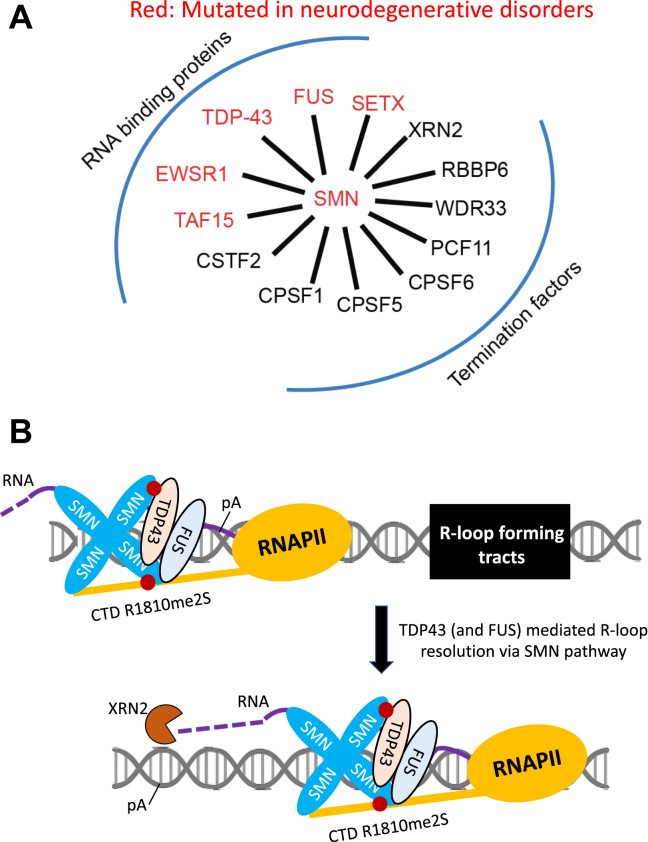
**Proposed model for the role of TDP-43 and FUS in transcription termination**. SMN as a nucleator of arginine dimethylated proteins involved in transcription termination and neurodegeneration (*A*), through the recruitment of FUS or TDP-43 to prevent R-loop formation and DNA damage at RNAPII termination sites (*B*). *Red dot*: arginine dimethylation.

SMN is a multifunctional protein implicated in numerous aspects of RNA metabolism, including pre-mRNA splicing, translation, RNA trafficking, histone mRNA processing, and stress granule dynamics (68). SMN is also involved in DNA repair, transcriptional regulation, signal transduction, and cytoskeletal organization (69). These diverse functions are largely facilitated by SMN’s ability to self-associate and to interact with a wide array of ribonucleoprotein (RNP) complexes, the dysfunction of which underlies the neuromuscular pathology observed in SMA (12, 13, 70). We previously demonstrated that SMN interacts with symmetrically dimethylated arginines in the C-terminal domain (CTD) of RNA polymerase II and functions in targeting the SETX protein to R-loops (11). Building upon this, we have now provided evidence that SMN directly or indirectly interacts with FUS and TDP-43, in addition to several transcription termination factors harboring dimethylated arginines, such as XRN2, and may function as a molecular scaffold that coordinates the assembly of an R-loop resolution complex. Our findings suggest a model wherein SMN interacts with FUS and TDP-43 to promote RNAPII termination by resolving R-loops (Fig. 8, *A* and *B*). This complex may be especially critical for gene terminators rich in G-quadruplex-forming sequences, which are prone to R-loop formation and genomic instability (71).

The ability of SMN to interact with multiple R-loop resolution proteins that are also genetically linked to neurodegenerative diseases (*e*.*g*., FUS and TDP-43, which also interact with each other) strengthens the emerging view that ALS and SMA share overlapping molecular etiologies (52, 53, 72). Indeed, of the ∼150 human proteins that have been shown by mass spectrometry to contain dimethylated arginine, over one-third have been implicated in the RNAPII transcription and termination processes, including core termination and elongation factors such as FUS, TDP-43, EWSR1, TAF15, CPSF1, CPSF5, CPSF6, SPT5, CTDP1, PABP1, and PABP2 (18, 19, 20, 21, 22). Given the prevalence of dimethylated arginine modifications among these factors, the role of SMN in orchestrating the RNP landscape during transcription termination deserves further investigation. Existing evidence indicates that SMN Tudor domain binding to Rme2s-containing substrates is generally in the micromolar to millimolar range (73, 74). Moreover, the dissociation constant (Kd) for SMN Tudor binding to the RNAPII CTD R1810me2s have been reported in the micromolar range (75). These relatively weak interactions are consistent with the SMN Tudor domain engaging a broad range of substrates. Although we did not determine dissociation constants for the interactions among TDP-43, FUS, SMN, and RNAPII, our co-immunoprecipitation experiments nevertheless demonstrate robust interaction in cells, consistent with *in vivo* enhancement of binding through SMN oligomerization, multivalent interactions, or high local substrate concentration. Future biochemical reconstitution experiments will be required to define precise Kd values for these interactions.

In addition to the canonical interactions of the Tudor domain of SMN with dimethylated arginine residues, the intrinsically disordered regions (IDRs) of TDP-43, FUS, and SMN may also contribute to complex formation. IDRs can engage in weak, multivalent interactions that enhance binding and potentially facilitate the assembly of protein-RNA complexes or phase-separated condensates (76). While our current experiments do not specifically dissect the contributions of IDRs, the robust co-immunoprecipitation of these proteins from cell extracts suggests that IDRs may help stabilize these interactions *in vivo*. Notably, the SMN Tudor domain preferentially recognizes symmetrically dimethylated arginine motifs within RG/RGG-rich sequences (77), and such RG/RGG repeats are embedded within the IDRs of both TDP-43 and FUS. Future studies using IDR-deletion or domain-specific mutants will be required to quantitatively assess how IDRs contribute to the assembly, stability, and regulation of TDP-43–FUS–SMN complexes.

The role of RNA-binding proteins in transcription termination and regulation of alternative cleavage and polyadenylation is well documented in both earlier reports and many recent studies (78, 79, 80, 81, 82, 83, 84). While our findings describing the role of TDP-43 in R-loop resolution through RNA-binding were available in preprint (85), two recent reports provided evidence to support TDP-43’s role in R-loop resolution. Giannini *et al*. (2020) showed that nuclear depletion of TDP-43 leads to a marked increase in genotoxic R-loops and the accumulation of FANCD2 foci, suggesting impaired R-loop clearance (86). Similarly, Wood *et al*. (2020) demonstrated that TDP-43 preserves replication fork progression by regulating R-loop formation through a transcription-coupled mechanism (62). These findings and our results reported here are consistent with observations that TDP-43 associates with RNAPII and regulates transcription (87, 88), thus placing it in proximity to nascent transcripts and R-loop-prone sites. More recently, it was demonstrated that chronic deficiency of TDP-43 alters the crosstalk between R-loops and 5-hydroxymethylcytosine (5hmC) in gene bodies and long-range enhancer/promoter interactions (89), further highlighting the role of TDP-43 in R-loop homeostasis. Moreover, recent studies have also highlighted a role for FUS’s RNA-binding activity in preventing R-loop formation and in associating with alternative polyadenylation sites, where it regulates transcription termination by stalling RNAPII (56, 90, 91). In addition to their roles in termination, both FUS and TDP-43 have been implicated in regulating gene expression more broadly. TDP-43, for instance, influences the transcription of both protein-coding genes and repetitive elements such as Alu sequences (88), although the mechanisms remain poorly defined. The loss of TDP-43 nuclear functions has also been linked to genomic instability in ALS neurons (92). The potential of TDP-43 to compete with other termination factors at UG-rich motifs downstream of the polyadenylation signal suggests a nuanced regulatory role that may impact transcript isoform diversity and genome stability (93, 94, 95). FUS associates with the RNAPII CTD and regulates its Ser2 phosphorylation status, thereby affecting cleavage/polyadenylation and promoting efficient termination (29, 56). Furthermore, both FUS and TDP-43 suppress the buildup of transcription-associated DNA damage (55, 96, 97, 98, 99), underscoring their broad roles as genome guardians.

The accumulation of R-loops is particularly problematic at GC- or G-rich genomic tracts (17, 100)—features commonly found in promoters, terminators, and disease-associated trinucleotide repeat expansions, such as those in ALS/FTD, Fragile X syndrome, and Friedreich’s Ataxia (101, 102, 103, 104). Such regions are inherently prone to R-loop formation and are frequent sites of transcription-replication conflicts (47, 71, 105). In the absence of proper resolution, R-loops at these loci can trigger DNA double-strand breaks, mutations, and chromosome instability (64, 67, 106). Indeed, their stabilization is observed when key factors involved in transcription-coupled nucleotide excision repair (63), homologous recombination (107), or the Fanconi anemia pathway (108, 109) are depleted. As a result, eukaryotic cells have evolved multiple R-loop surveillance pathways to maintain genomic integrity (46, 110). In this regard, our results place SMN, TDP-43, and FUS as critical regulators of R-loops in transcription termination regions.

It is increasingly clear that defective RNA processing and surveillance, especially involving RNA-binding proteins such as FUS and TDP-43, play a central role in ALS pathogenesis (111, 112). The ability of these proteins to bind RNA and prevent pathological R-loop formation may explain our observation that loss of either factor leads to RNAPII stalling and accumulation at transcription terminators. Motor neurons may be particularly vulnerable to dysfunction of TDP-43 (and FUS) because neurons in general, and motor neurons in particular, tend to express a very large proportion of long genes. These long genes are intrinsically prone to transcriptional stress, R-loop formation, and defects in transcription termination. Multiple studies have shown that neuronal transcriptomes are enriched for genes that are more than 100 kb in length, and many of these genes encode synaptic, cytoskeletal, and axonal maintenance factors, which are critical for motor-neuron function (113). If R-loop resolution and normal termination are compromised, as suggested by our findings on TDP-43 and FUS function, long genes are likely to be disproportionately affected. This may result in enhanced RNAPII stalling, R-loop accumulation, and increased susceptibility to transcription-associated DNA damage. R-loop dysregulation has been widely linked to neurodegeneration and motor-neuron disease (114). In addition, post-mitotic neurons have limited capacity for error-free DNA repair, and increased DNA damage has been documented in human ALS motor neurons (97, 115). Together, these observations support the possibility that motor neurons cross a threshold of accumulated transcription stress and insufficient repair when TDP-43 or FUS function is impaired, which could explain their selective vulnerability. Given that ALS and FTD often manifest late in life, we hypothesize that the gradual accumulation of unresolved R-loops and the resulting DNA damage in post-mitotic neurons contributes significantly to the onset and progression of neurodegeneration. Our findings highlight the critical function of the SMN–FUS–TDP-43 axis in transcriptional homeostasis and underscore the importance of R-loop resolution as a shared vulnerability in both ALS and SMA.

## Experimental procedures

### Cell cultures

Flp-In 293 T-REx cell lines were obtained from Life Technologies (R780–07), and stable Flp-In 293 T-REx cell lines after the incorporation of the transgene were maintained with hygromycin (Life Technologies, 10687010) at 2ug/ml. Cell cultures were maintained in Dulbecco's modified Eagle's medium (DMEM) (Wisent Bioproducts catalogue number 319–005-CL) supplemented with 10% FBS (Wisent Bioproducts catalogue number 080–705), sodium pyruvate, non-essential amino acids, and penicillin/streptomycin, as described (116). Stably shRNA knocked down transduced Flp-In 293 T-REx cell lines were maintained with 2 μg/ml puromycin (Sigma, p8833). Raji cells were cultured in RPMI (SLRI media facility, University of Toronto) plus 10% Tetracycline free FBS (Gibco) and 1% Glutamate, and stably transduced cells were maintained with 500 μg/ml G418 (Gibco, 11811031). There was no evidence of *mycoplasma* contamination of the cell lines used in this work as judged by staining of fixed cells with DAPI.

### shRNA knockdowns, siRNA knockdowns, CRISPR/Cas9 knockouts, and GFP overexpression

shRNAs in lentivirus vectors were used to stably transduce cell lines using an established protocol (117). siRNA knockdowns for HEK293 cells were performed with 50 nM siRNAs with PepMute siRNA transfection reagent (SigmaGen Laboratory, SL100566) for 3 days siRNAs against human FUS (NM_001010850) and TDP-43 (NM_007375) and scrambled control were purchased from Sigma. For CRISPR/Cas9-mediated gene knockouts, CRISPR/Cas9 plasmids (pCMV-Cas9-GFP) were purchased from Sigma-Aldrich to express the scrambled guide RNA, or guide RNAs for the KO of TDP-43 or FUS. 2 μg of the plasmids were transfected into HEK293 cells, and 1 day after lipofectamine 3000 transfection, cells were sorted by BD FACSAria flow cytometry (Donnelly Centre, University of Toronto) and single GFP-positive cells were plated and expanded in a 48-well plate. The expression level of TDP-43 or FUS in each clone was detected by western blotting.

For CRISPR/Cas9-mediated knock-in mutation of POLR2A R1810 to alanine, we used CRISPR/Cas9 plasmids (pCMV-Cas9-GFP) expressing the scrambled guide RNA, or guide RNAs targeting the 29th exon of POLR2A with the following sequences: 5′-CACCGGAGACTGTGGTGTGTATCGT-3′; 5′-AAACACGATACACACCACAGTCTCC-3′. We synthesized a repair DNA template of 750 base pairs (Life Technologies) that contained the desired mutation of CGA → GCA to create the R1810 → A mutation. 1 μg of the CRISPR/Cas9 plasmids and 3ug of the repair template were transfected into HEK293 cells, and 2 days after lipofectamine 3000 transfection, cells were sorted by BD FACSAria flow cytometry and single cells were plated and expanded into 96-well plates. The R1810 A mutation was verified by sequencing cDNA extracted from cells derived from the individual clones.

For the stable over-expression of the TDP-43-GFP in HEK293 cells, wild-type TDP-43 ORF (Harvard Plasmid database, HsCD00079870) was cloned into the pDEST pcDNA5/FRT/TO-eGFP through Gateway cloning (ThermoFisher). The truncation mutant (GFP-TDP-43 RRM-) lacking the RRM coding (S104-P262) residues was generated from the wild-type ORF template through PCR splicing/overlap extension PCR; which employs two complementary primers that comprise a fused sequence of −15 bp to +15 bp related to the junction point (5′-GCAGTCCAGAAAACA-3′+5′-AAGCACAATAGCAAT-3′) in multiple PCR reactions with a high fidelity Taq DNA polymerase (ThermoFisher, 11304011). The truncated ORF was then cloned into the pDEST pcDNA5/FRT/TO-eGFP through Gateway cloning.

### Cell transfection and electroporation

Raji cells with stable expression of HA-tagged wild-type or R1810 A POLR2A constructs were generated by electroporation (10 μg of plasmid DNA per 10^7^ cells) followed by selection and maintenance in G418 (0.5 mg/ml). α-amanitin treatment was carried out with 2 μg/ml α-amanitin for 3 days for Co-IP and ChIP experiments involving HA-tagged wild type or R1810 A POLR2A.

The transfections of the CRISPR/Cas9 plasmids (pCMV-Cas9-GFP) and GFP-tagged (pDEST pcDNA5/FRT/TO-eGFP) transgene into the Flp-In 293 T-REx cell lines were performed with FuGENE Transfection Reagent (Roche, E269 A).

### Immunostaining

For immunostaining, cells were fixed with 4% paraformaldehyde (Sigma, P6148) and washed with PBS + 0.1% Triton X 100 (Sigma, T8532). Primary antibodies 1:50-1:100 in PBST + 30 mg/ml BSA (Roche, 10735108001) were added to cells for staining overnight at 4 °C. Cells were washed with PBST and stained with Alexa Fluor 488 or 594 (Invitrogen) at 1:1000 and Hoechst 33342 (Thermo Scientific) in PBST with 5% goat serum (Sigma, G9023) at room temperature for 1 h, followed by washes with PBS + 0.2% Triton and mounting with ImmunoMount (GeneTex, GTX30928). Images were taken using an Olympus Upright Microscope BX61 with Optigrid function and processed using Volocity OpenLab Software (PerkinElmer).

### Immunoprecipitation (IP) and Western blots

IP was performed with RIPA buffer (140 mM NaCl, 10 mM Tris pH7.6–8, 1% Triton, 0.1% Na deoxycholate, 1 mM EDTA) containing protease inhibitors (Roche, 05892791001) and Benzonase (Sigma, E1014), as previously described (118). 1-2x10^7^ cells were lysed on ice for 25 min by vortexing and forcing them through a 27-gauge needle. After centrifuging at 13,000 rpm for 10 min at 4 °C, the supernatant was incubated with 25 μl (1:10 dilution) protein G beads (Invitrogen, 10–1243, 10003D) and 1-2 μg of antibodies for 4 h to overnight. The samples were washed 3 times with RIPA buffer and boiled in SDS gel sample buffer. To detect R1810me2s or R1810me2a modifications on POLR2A, alkaline phosphatase (Roche, 10108138001) treatment (5 μl) at 37 °C for 30 min was performed for POLR2A IP samples before boiling. Samples were run using 7.5 to 10% SDS-PAGE (and 4–12% BIS-TRIS PAGE) and transferred to PVDF membranes (Bio-Rad, 162–0177) using a Trans-Blot SD Semi-Dry Electrophoretic Transfer Cell (BioRad, 170–3940). Primary antibodies were used at 1:250 to 1:1000 dilutions for incubation overnight, and horseradish peroxidase-conjugated goat anti-mouse, -rabbit, or –rat secondary antibodies were used at 1:10,000 (Dako, P0450). Blots were developed using SuperSignal West Pico or Femto (Thermo Scientific, 34079, 34094). Co-IP were performed with ≥ 2 biological replicates.

Protein bands on blots (low exposure versions for linear range) were quantified by densitometry (ImageJ software). Band intensities were background-subtracted and IgG control signal subtracted. For each IP lane, prey band intensity was normalized to the amount of bait recovered in the same lane (Normalized binding = (IP_prey − IgG_prey)/(IP_bait − IgG_bait)). For background, we measured an adjacent region of the blot without bands (same box size). To calculate %INP, we used the following formula %Input = (Adj_IP_prey/INP_prey) ∗ fraction_input_loaded ∗ 100, where Adj_IP_prey = IP_prey - IgG_prey.

### Chromatin immunoprecipitation (ChIP) and R-loop detection

Chromatin immunoprecipitation (ChIP) was performed using the EZ-ChIP A-Chromatin Immunoprecipitation Kit (Millipore, 17–371) or similar homemade solutions according to the manufacturer’s instructions. Antibodies were used in the 1-2 μg range, and IgG was used as a background control. R-loop detection was performed according to El Hage *et al*. (2010) with minor modifications (106). R-loop detection was performed following the ChIP protocol except that, after the nuclear lysis and sonication, genomic DNA was de-crosslinked in ChIP elution buffer containing 5M NaCl at 65 °C overnight. DNA was purified with the Qiaex II kit (Qiagen, 20021) for PCR product purification and eluted in water. S9.6 binding was carried out overnight with 25 μl of Dynabeads protein G beads (Invitrogen, 100–03D) and 1 μg of antibody purified from the S9.6 hybridoma cell line (119) that recognizes RNA/DNA hybrids. Immunoprecipitated and input DNAs were used as templates for qPCR. RNase-sensitivity analysis for R-loops was carried out by adding 50 U of RNase H (Invitrogen, 18021–014) in 10X RNase H buffer (75 mM KCl, 50 mM Tris pH8.3, 3 mM MgCl2, 10 mM DTT) with 4% glycerol and 20 μg/ml BSA prior to immunoprecipitation; the RNase H treatment was performed for 2 h at 37 °C.

For comparing POLR2A and S9.6 signals on the *ACTB* gene, wild type or control signals were normalized to 1, and the R1810A mutant, knockdown or knock-out samples were adjusted such that the ratio for the intron 3 (1671) position was set to 1. Similarly, for the *GAPDH* gene, the ratio for the intron 5 (2436) position was set to 1. ChIP data for SMN, FUS, and TDP-43 were expressed as percent input or as ratio to the ChIP data for POLR2A. Error bars represent biological replicates, except where indicated otherwise.

### Primer information

Primers used in ChIP are listed here. For the *ACTB* gene:

−72.fw CCGAAAGTTGCCTTTTATGGC, −72.rev CAAAGGCGAGGCTCTGTGC;

332.fw CGGGGTCTTTGTCTGAGC, 332.rev CAGTTAGCGCCCAAAGGAC;

1671.fw TAACACTGGCTCGTGTGACAA, 1671.rev AAGTGCAAAGAACACGGCTAA; 2911.fw TGCGCAGAAAACAAGATGAG, 2911.rev GTCACCTTCACCGTTCCAGT;

3560.fw TTACCCAGAGTGCAGGTGTG, 3560.rev CCCCAATAAGCAGGAACAGA;

3752.fw GGGACTATTTGGGGGTGTCT, 3752.rev TCCCATAGGTGAAGGCAAAG;

4657.fw TGGGCCACTTAATCATTCAAC, 4657.rev CCTCACTTCCAGACTGACAGC;

5590.fw CAGTGGTGTGGTGTGATCTTG, 5590.rev GGCAAAACCCTGTATCTGTGA.

For the *GAPDH* gene: 55.fw CTCCTGTTCGACAGTCAGC, 55.rev TTCAGGCCGTCCCTAGC; 1407.fw CACCCTGGTCTGAGGTTAAATATAG, 1407.rev GTGGGAGCACAGGTAAGT;

2436.fw ATAGGCGAGATCCCTCCAA, 2436.rev TGAAGACGCCAGTGGAC;

3882.fw CCCTGTGCTCAACCAGT, 3882.rev CTCACCTTGACACAAGCC;

4511.fw AGATGTGTCAGGGTGACTTAT, 4511.rev TAGGTCCCAGCTACACGC;

5196.fw GTCTCAGTGTATGACAGACACG, 5196.rev TGTATGTGCGCTCAGGG.

### ChIP-seq experiments

Chromatin immunoprecipitation was performed as before (120). In brief, 10^7^-10^8^ cells were cross-linked for 10 min in 1% formaldehyde. Lysates were sonicated to a DNA fragment length range of 200 to 300 bp using a Bioruptor (Diagenode). RNAPII was immunoprecipitated with 2 μg of antibodies and Dynabeads Protein G (Invitrogen). Subsequently, crosslinks were reversed at 65 °C overnight and bound DNA fragments were purified (EZ-10 Spin Column PCR Product Purification kit, Bio Basic). Sequencing libraries were constructed using the TruSeq ChIP Sample Prep Kit (Illumina) according the manufacturer's instructions. Libraries were sequenced (single end reads) on the Illumina HiSeq 2500 to a minimum depth of 20 million reads, obtaining at least 12 to 20 million unique reads per sample.

### Antibodies, constructs, and reagents

Anti-CTD R1810me2s antibody was raised in rabbits using a KLH-conjugated CTD peptide from POLR2A (amino acids 1806–1813) that carried an R1810me2s modification. KLH conjugation was performed using an N-terminal cysteine residue (Cedarlane), as described in our previous report (11). R1810me2s-specific antibodies were enriched by flowing the serum through a column containing an R1810me0 peptide conjugated to SulfoLink coupling resin (Thermo Scientific, 20401).

Flp-in TREx GFP-HB fusion construct that contains the R-loop binding domain of RNase H was provided by Dr Andres Aguilera (66). The ORFs for TDP-43 came from the Plasmid Collection at Harvard. GFP constructs were generated *via* Gateway cloning into pDEST pcDNA5/FRT/TO-eGFP. α-amanitin-resistant wild-type and R1810A mutant POLR2A constructs were kindly provided by Dr Dirk Eick (58); RNAPII R1810me2a antibody was kindly provided by Dr Danny Reinberg (58). We obtained the POLR2A pSer2 and pSer5 antibodies from the Eick laboratory (S2P: 3E10; S5P: 3E8). 8WG16 antibody against unphosphorylated CTD repeats of POLR2A was prepared in the lab as reported previously (11).

Commercial antibodies were as follows: HA (Sigma, mAb H9658); PRMT5 (Upstate, pAb C7-405, Santa Cruz, mAb sc-22132); SMN (Santa Cruz Biotechnology, sc-32313; pAb H-195); SETX for ChIP and IP (Novus Biologicals, pAb NB100–57543) and for western blots (Bethyl Lab, pAb A301–104A); XRN2 (Santa Cruz, pAb sc-99237); FUS (Santa Cruz, mAb sc-47711); TDP-43 (Bethyl lab, pAb A303–233A); POLR2A N20 (Santa Cruz, pAb sc-899); POLR2A 4H8 (Abcam, mAb ab5408); gammaH2Ax (Millipore, 05–636); H2Ax (Millipore, 07–627); Tubulin (Sigma, mAb T8328); GAPDH (Santa Cruz, (0411): sc-47724);GFP (Abcam, pAb 290); and IgG negative controls for ChIP and IP (Millipore, pAb 12–370). α-amanitin was purchased from Sigma (23109–05–9).

### Quantification and statistical analysis

#### ChIP-seq analysis

ChIP-seq analysis was performed as before for the display of meta-gene plots (11). Reads in FASTQ format were mapped to the human genome (hg19) using Bowtie 2 (121) with options −5 1 to 3 2 –local duplicate reads removed, and reads were extended to 300 bp. The number of fragments overlapping each genomic bp was calculated and normalized by million mappable reads in the ChIP-seq library. Only the top four or 10% of genes by mRNA expression levels were considered.

For the calculating of promoter stalling and termination stopping ratios, signal density was calculated using the SPP (122) R package (bandwidth 150 bp, step 50 bp). Background from input was first scaled based on sequencing depth (total number of unique reads) and then subtracted from sample signal. Signal density in different experimental groups was quantile normalized to allow direct comparison between mutants and wild-type controls. Promoter region was defined as −30 bp to + 300 bp around the transcription start site. The rest (from +301 to the end of annotated gene) was defined as the gene body. Termination region was defined as the 2000-bp segment downstream from the polyA cleavage site. Signals in each of these regions were summed up and divided by the length of the region to get a normalized value. Promoter stalling ratio was defined as the ratio of promoter region signal over gene body signal, and termination stopping ratio was defined as the ratio of termination region signal over gene body signal. Only genes with signal in the gene body greater than one were included in the analysis. Quantiles of promoter stalling and termination stopping ratios were computed for KO mutants and control separately and plotted against increasing level of ratios. Statistically significant differences between the quantiles were determined using the Kolmogorov Smirnov test; level of significance was set at *p* < 0.05. Only the top 10% genes (∼2000 genes) by mRNA expression levels were considered; statistical analysis and data plotting were performed using R.

We analyzed TDP-43 ChIP-seq data in HEK293 T cells, which was made available by the ENCODE consortium (GSE92026) (59). The plots were generated using deeptools (123), and motif enrichment analysis was performed using DREME (124). DREME (version 5.3.2) was run with the following parameters: dreme -p TDP43_ChIP_conservative_peaks_slop5b.bed.fa -norc -k 6 -m 3 -rna -oc dreme_TDP43_ChIP_conservative_peaks_noInput.

Processed R-loop sequencing data (*i*.*e*., S9.6-based DNA/RNA IP (DRIP) sequencing) were acquired from a previously published study to identify R-loop positions in HEK293 cells (GEO: GSE68953) (61).

#### iCLIP-seq analysis

TDP-43 iCLIP-seq data were acquired from our recently published large-scale study of RNA-binding proteins in HEK293 cells (GEO: GSE230846) (60). Analysis of iCLIP-seq data was performed essentially as previously described (125, 126). Briefly, 51-nt raw reads that consisted of three random positions, a 4-nt multiplexing barcode, and another two random positions, followed by the cDNA sequence, were de-duplicated based on the first 45 nt. Reads were de-multiplexed, and the random positions, barcodes, and any 3′-bases matching Illumina adaptors were removed. Finally, reads shorter than 25 nt were removed and the remaining reads were trimmed to 35 nt using Trimmomatic (127). Reads were then mapped to the human genome/transcriptome (Ensembl annotation of hg19) using Tophat (128) with default settings. Reads with a mapping quality < 3 were removed from further analysis.

Metagene plots and peak distribution analysis across genomic regions (analyzing 5′ and 3′UTRs separately) were generated using the R package GenomicPlot (URL: https://github.com/shuye2009/GenomicPlot), as reported in our previous study (60). Motif enrichment analysis was performed using DREME pipeline to identify motifs that are associated with TDP-43 crosslink sites (124).

## Data availability

Data for ChIP-seq analyses have been deposited in GEO with the accession code GSE134332.

## Supporting information

This article contains supporting information.

## Conflict of interest

The authors declare that they do not have any conflicts of interest with the content of this article.

## References

[1] Buratowski S. (2009). Progression through the RNA polymerase II CTD cycle. Mol. Cell..

[2] Egloff S., Murphy S. (2008). Role of the C-terminal domain of RNA polymerase II in expression of small nuclear RNA genes. Biochem. Soc. Trans..

[3] Hengartner C.J., Myer V.E., Liao S.M., Wilson C.J., Koh S.S., Young R.A. (1998). Temporal regulation of RNA polymerase II by Srb10 and Kin28 cyclin-dependent kinases. Mol. Cell..

[4] Komarnitsky P., Cho E.J., Buratowski S. (2000). Different phosphorylated forms of RNA polymerase II and associated mRNA processing factors during transcription. Genes Dev..

[5] Larochelle S., Amat R., Glover-Cutter K., Sansó M., Zhang C., Allen J.J. (2012). Cyclin-dependent kinase control of the initiation-to-elongation switch of RNA polymerase II. Nat. Struct. Mol. Biol..

[6] Pilarova K., Herudek J., Blazek D. (2020). CDK12: cellular functions and therapeutic potential of versatile player in cancer. NAR Cancer.

[7] Tellier M., Zaborowska J., Caizzi L., Mohammad E., Velychko T., Schwalb B. (2020). CDK12 globally stimulates RNA polymerase II transcription elongation and carboxyl-terminal domain phosphorylation. Nucleic Acids Res..

[8] Blazek D., Kohoutek J., Bartholomeeusen K., Johansen E., Hulinkova P., Luo Z. (2011). The cyclin K/Cdk12 complex maintains genomic stability via regulation of expression of DNA damage response genes. Genes Dev..

[9] Davidson L., Muniz L., West S. (2014). 3’ end formation of pre-mRNA and phosphorylation of Ser2 on the RNA polymerase II CTD are reciprocally coupled in human cells. Genes Dev..

[10] Eifler T.T., Shao W., Bartholomeeusen K., Fujinaga K., Jäger S., Johnson J.R. (2014). Cyclin-dependent kinase 12 increases 3′ end processing of growth factor-induced c-FOS transcripts. Mol. Cell Biol..

[11] Yanling Zhao D., Gish G., Braunschweig U., Li Y., Ni Z., Schmitges F.W. (2016). SMN and symmetric arginine dimethylation of RNA polymerase II C-terminal domain control termination. Nature.

[12] Burghes A.H.M., Beattie C.E. (2009). Spinal muscular atrophy: why do low levels of survival motor neuron protein make motor neurons sick?. Nat. Rev. Neurosci..

[13] Martin R., Gupta K., Ninan N.S., Perry K., Van Duyne G.D. (2012). The survival motor neuron protein forms soluble glycine zipper oligomers. Structure.

[14] Lorson C.L., Strasswimmer J., Yao J.M., Baleja J.D., Hahnen E., Wirth B. (1998). SMN oligomerization defect correlates with spinal muscular atrophy severity. Nat. Genet..

[15] Tsui A., Kouznetsova V.L., Kesari S., Fiala M., Tsigelny I.F. (2023). Role of senataxin in amyotrophic lateral sclerosis. J. Mol. Neurosci..

[16] Suraweera A., Lim Y.C., Woods R., Birrell G.W., Nasim T., Becherel O.J. (2009). Functional role for senataxin, defective in ataxia oculomotor apraxia type 2, in transcriptional regulation. Hum. Mol. Genet..

[17] Skourti-Stathaki K., Proudfoot N.J., Gromak N. (2011). Human senataxin resolves RNA/DNA hybrids formed at transcriptional pause sites to promote Xrn2-dependent termination. Mol. Cell.

[18] Boisvert F.M., Côté J., Boulanger M.C., Richard S. (2003). A proteomic analysis of arginine-methylated protein complexes. Mol. Cell Proteomics.

[19] Uhlmann T., Geoghegan V.L., Thomas B., Ridlova G., Trudgian D.C., Acuto O. (2012). A method for large-scale identification of protein arginine methylation. Mol. Cell Proteomics.

[20] Guo A., Gu H., Zhou J., Mulhern D., Wang Y., Lee K.A. (2014). Immunoaffinity enrichment and mass spectrometry analysis of protein methylation. Mol. Cell Proteomics.

[21] Boisvert F.M., Chénard C.A., Richard S. (2005). Protein interfaces in signaling regulated by arginine methylation. Sci. STKE.

[22] Shi Y., Di Giammartino D.C., Taylor D., Sarkeshik A., Rice W.J., Yates J.R. (2009). Molecular architecture of the human pre-mRNA 3’ processing complex. Mol. Cell.

[23] Musiani D., Bok J., Massignani E., Wu L., Tabaglio T., Ippolito M.R. (2019). Proteomics profiling of arginine methylation defines PRMT5 substrate specificity. Sci. Signal.

[24] Al-Chalabi A., Jones A., Troakes C., King A., Al-Sarraj S., Van Den Berg L.H. (2012). The genetics and neuropathology of amyotrophic lateral sclerosis. Acta Neuropathol..

[25] Kwiatkowski T.J., Bosco D.A., LeClerc A.L., Tamrazian E., Vanderburg C.R., Russ C. (2009). Mutations in the FUS/TLS gene on chromosome 16 cause familial amyotrophic lateral sclerosis. Science (1979).

[26] Vance C., Rogelj B., Hortobágyi T., De Vos K.J., Nishimura A.L., Sreedharan J. (2009). Mutations in FUS, an RNA processing protein, cause familial amyotrophic lateral sclerosis type 6. Science (1979).

[27] Sreedharan J., Blair I.P., Tripathi V.B., Hu X., Vance C., Rogelj B. (2008). TDP-43 mutations in familial and sporadic amyotrophic lateral sclerosis. Science (1979).

[28] Kabashi E., Valdmanis P.N., Dion P., Spiegelman D., McConkey B.J., Velde C.V. (2008). TARDBP mutations in individuals with sporadic and familial amyotrophic lateral sclerosis. Nat. Genet..

[29] Schwartz J.C., Ebmeier C.C., Podell E.R., Heimiller J., Taatjes D.J., Cech T.R. (2012). FUS binds the CTD of RNA polymerase II and regulates its phosphorylation at Ser2. Genes Dev..

[30] Lagier-Tourenne C., Polymenidou M., Hutt K.R., Vu A.Q., Baughn M., Huelga S.C. (2012). Divergent roles of ALS-linked proteins FUS/TLS and TDP-43 intersect in processing long pre-mRNAs. Nat. Neurosci..

[31] Hallegger M., Chakrabarti A.M., Lee F.C.Y., Lee B.L., Amalietti A.G., Odeh H.M. (2021). TDP-43 condensation properties specify its RNA-binding and regulatory repertoire. Cell.

[32] Cooper-Knock J., Walsh M.J., Higginbottom A., Highley J.R., Dickman M.J., Edbauer D. (2014). Sequestration of multiple RNA recognition motif-containing proteins by C9orf72 repeat expansions. Brain.

[33] Mori K., Weng S.M., Arzberger T., May S., Rentzsch K., Kremmer E. (2013). The C9orf72 GGGGCC repeat is translated into aggregating dipeptide-repeat proteins in FTLD/ALS. Science (1979).

[34] Bruijn L.I., Houseweart M.K., Kato S., Anderson K.L., Anderson S.D., Ohama E. (1998). Aggregation and motor neuron toxicity of an ALS-linked SOD1 mutant independent from wild-type SOD1. Science (1979).

[35] Mackenzie I.R.A., Rademakers R., Neumann M. (2010). TDP-43 and FUS in amyotrophic lateral sclerosis and frontotemporal dementia. Lancet Neurol..

[36] Kawaguchi T., Rollins M.G., Moinpour M., Morera A.A., Ebmeier C.C., Old W.M. (2020). Changes to the TDP-43 and FUS interactomes induced by DNA damage. J. Proteome Res..

[37] Demongin C., Tranier S., Joshi V., Ceschi L., Desforges B., Pastré D. (2024). RNA and the RNA-binding protein FUS act in concert to prevent TDP-43 spatial segregation. J. Biol. Chem..

[38] Jo M., Lee S., Jeon Y.M., Kim S., Kwon Y., Kim H.J. (2020). The role of TDP-43 propagation in neurodegenerative diseases: integrating insights from clinical and experimental studies. Exp. Mol. Med..

[39] Sama R.R.K., Ward C.L., Bosco D.A. (2014). Functions of FUS/TLS from DNA repair to stress response: implications for ALS. ASN Neuro.

[40] Vance C., Scotter E.L., Nishimura A.L., Troakes C., Mitchell J.C., Kathe C. (2013). ALS mutant FUS disrupts nuclear localization and sequesters wild-type FUS within cytoplasmic stress granules. Hum. Mol. Genet..

[41] Janssens J., Van Broeckhoven C. (2013). Pathological mechanisms underlying TDP-43 driven neurodegeneration in FTLD-ALS spectrum disorders. Hum. Mol. Genet..

[42] Chatterjee M., Özdemir S., Fritz C., Möbius W., Kleineidam L., Mandelkow E. (2024). Plasma extracellular vesicle tau and TDP-43 as diagnostic biomarkers in FTD and ALS. Nat. Med..

[43] Dormann D., Rodde R., Edbauer D., Bentmann E., Fischer I., Hruscha A. (2010). ALS-associated fused in sarcoma (FUS) mutations disrupt transportin-mediated nuclear import. EMBO J..

[44] Neumann M., Sampathu D.M., Kwong L.K., Truax A.C., Micsenyi M.C., Chou T.T. (2006). Ubiquitinated TDP-43 in frontotemporal lobar degeneration and amyotrophic lateral sclerosis. Science.

[45] Sanz L.A., Hartono S.R., Lim Y.W., Steyaert S., Rajpurkar A., Ginno P.A. (2016). Prevalent, dynamic, and conserved R-Loop structures associate with specific epigenomic signatures in mammals. Mol. Cell.

[46] Aguilera P., Aguilera A. (2025). R-loop homeostasis in genome dynamics, gene expression and development. Curr. Opin. Genet. Dev..

[47] Skourti-Stathaki K., Kamieniarz-Gdula K., Proudfoot N.J. (2014). R-loops induce repressive chromatin marks over mammalian gene terminators. Nature.

[48] García-Muse T., Aguilera A., Loops R. (2019). From physiological to pathological roles. Cell..

[49] Luna R., Gómez-González B., Aguilera A. (2024). RNA biogenesis and RNA metabolism factors as R-loop suppressors: a hidden role in genome integrity. Genes Dev..

[50] Hernández-Reyes Y., Fonseca-Rodríguez C., Freire R., Smits V.A.J. (2025). DDX37 and DDX50 maintain genome stability by preventing transcription-dependent R-loop formation. J. Mol. Biol..

[51] Walker C., Herranz-Martin S., Karyka E., Liao C., Lewis K., Elsayed W. (2017). C9orf72 expansion disrupts ATM-mediated chromosomal break repair. Nat. Neurosci..

[52] Yamazaki T., Chen S., Yu Y., Yan B., Haertlein T.C., Carrasco M.A. (2012). FUS-SMN protein interactions link the motor neuron diseases ALS and SMA. Cell Rep..

[53] Groen E.J.N., Fumoto K., Blokhuis A.M., Engelen-Lee J.Y., Zhou Y., van den Heuvel D.M.A. (2013). ALS-associated mutations in FUS disrupt the axonal distribution and function of SMN. Hum. Mol. Genet..

[54] Ederle H., Funk C., Abou-Ajram C., Hutten S., Funk E.B.E., Kehlenbach R.H. (2018). Nuclear egress of TDP-43 and FUS occurs independently of Exportin-1/CRM1. Sci. Rep..

[55] Hill S.J., Mordes D.A., Cameron L.A., Neuberg D.S., Landini S., Eggan K. (2016). Two familial ALS proteins function in prevention/repair of transcription-associated DNA damage. Proc. Natl. Acad. Sci. U. S. A..

[56] Masuda A., Takeda J.I., Okuno T., Okamoto T., Ohkawara B., Ito M. (2015). Position-specific binding of FUS to nascent RNA regulates mRNA length. Genes Dev..

[57] Burke K.A., Janke A.M., Rhine C.L., Fawzi N.L. (2015). Residue-by-Residue view of In vitro FUS granules that bind the C-Terminal domain of RNA polymerase II. Mol. Cell.

[58] Sims R.J., Rojas L.A., Beck D., Bonasio R., Schüller R., Drury W.J. (2011). The C-terminal domain of RNA polymerase II is modified by site-specific methylation. Science (1979).

[59] Dunham I., Kundaje A., Aldred S.F., Collins P.J., Davis C.A., Doyle F. (2012). An integrated encyclopedia of DNA elements in the human genome. Nature.

[60] Nabeel-Shah S., Pu S., Burns J.D., Braunschweig U., Ahmed N., Burke G.L. (2024). C2H2-zinc-finger transcription factors bind RNA and function in diverse post-transcriptional regulatory processes. Mol. Cell..

[61] Nadel J., Athanasiadou R., Lemetre C., Wijetunga N.A., Ó Broin P., Sato H. (2015). RNA:DNA hybrids in the human genome have distinctive nucleotide characteristics, chromatin composition, and transcriptional relationships. Epigenetics Chromatin.

[62] Wood M., Quinet A., Lin Y.L., Davis A.A., Pasero P., Ayala Y.M. (2020). TDP-43 dysfunction results in R-loop accumulation and DNA replication defects. J. Cell Sci..

[63] Sollier J., Stork C.T., García-Rubio M.L., Paulsen R.D., Aguilera A., Cimprich K.A. (2014). Transcription-coupled nucleotide excision repair factors promote R-Loop-Induced genome instability. Mol. Cell.

[64] Aguilera A., García-Muse T., Loops R. (2012). From transcription byproducts to threats to genome stability. Mol. Cell.

[65] Gatti V., De Domenico S., Melino G., Peschiaroli A. (2023). Senataxin and R-loops homeostasis: multifaced implications in carcinogenesis. Cell Death Discov..

[66] Bhatia V., Barroso S.I., García-Rubio M.L., Tumini E., Herrera-Moyano E., Aguilera A. (2014). BRCA2 prevents R-loop accumulation and associates with TREX-2 mRNA export factor PCID2. Nature.

[67] Hatchi E., Skourti-Stathaki K., Ventz S., Pinello L., Yen A., Kamieniarz-Gdula K. (2015). BRCA1 recruitment to transcriptional pause sites is required for R-loop-driven DNA damage repair. Mol. Cell.

[68] Chaytow H., Huang Y.T., Gillingwater T.H., Faller K.M.E. (2018). The role of survival motor neuron protein (SMN) in protein homeostasis. Cell Mol. Life. Sci..

[69] Singh R.N., Howell M.D., Ottesen E.W., Singh N.N. (2017). Diverse role of survival motor neuron protein. Biochim. Biophys. Acta. Gene Regul Mech..

[70] Young P.J., Man N.T., Lorson C.L., Le T.T., Androphy E.J., Burghes A.H.M. (2000). The exon 2b region of the spinal muscular atrophy protein, SMN, is involved in self-association and SIP1 binding. Hum. Mol. Genet..

[71] Wulfridge P., Sarma K. (2024). Intertwining roles of R-loops and G-quadruplexes in DNA repair, transcription, and genome organization. Nat. Cell Biol..

[72] Chi B., O’Connell J.D., Iocolano A.D., Coady J.A., Yu Y., Gangopadhyay J. (2018). The neurodegenerative diseases ALS and SMA are linked at the molecular level via the ASC-1 complex. Nucleic Acids Res.

[73] Liu K., Guo Y., Liu H., Bian C., Lam R., Liu Y. (2012). Crystal structure of TDRD3 and methyl-arginine binding characterization of TDRD3, SMN and SPF30. PLoS One.

[74] Tripsianes K., Madl T., MacHyna M., Fessas D., Englbrecht C., Fischer U. (2011). Structural basis for dimethylarginine recognition by the tudor domains of human SMN and SPF30 proteins. Nat. Struct. Mol. Biol..

[75] Liu Y., Iqbal A., Li W., Ni Z., Wang Y., Ramprasad J. (2022). A small molecule antagonist of SMN disrupts the interaction between SMN and RNAP II. Nat Commun.

[76] Xiao L., Xia K. (2025). Functions of intrinsically disordered regions. Biology (Basel)..

[77] Friesen W.J., Massenet S., Paushkin S., Wyce A., Dreyfuss G. (2001). SMN, the product of the spinal muscular atrophy gene, binds preferentially to dimethylarginine-containing protein targets. Mol. Cell.

[78] Lovci M.T., Ghanem D., Marr H., Arnold J., Gee S., Parra M. (2013). Rbfox proteins regulate alternative mRNA splicing through evolutionarily conserved RNA bridges. Nat. Struct. Mol. Biol..

[79] Ni Z., Ahmed N., Nabeel-Shah S., Guo X., Pu S., Song J. (2024). Identifying human pre-mRNA cleavage and polyadenylation factors by genome-wide CRISPR screens using a dual fluorescence readthrough reporter. Nucleic Acids Res..

[80] Dharmalingam P., Mahalingam R., Yalamanchili H.K., Weng T., Karmouty-Quintana H., Guha A. (2022). Emerging roles of alternative cleavage and polyadenylation (APA) in human disease. J Cell Physiol..

[81] Liu X., Xie H., Liu W., Zuo J., Li S., Tian Y. (2024). Dynamic regulation of alternative polyadenylation by PQBP1 during neurogenesis. Cell Rep..

[82] Ashfield R., Patel A.J., Bossone S.A., Brown H., Campbell R.D., Marcu K.B. (1994). MAZ-dependent termination between closely spaced human complement genes. EMBO J..

[83] Rosonina E., Kaneko S., Manley J.L. (2006). Terminating the transcript: breaking up is hard to do. Genes Dev..

[84] Calvo O., Manley J.L. (2001). Evolutionarily conserved interaction between CstF-64 and PC4 links transcription, polyadenylation, and termination. Mol. Cell.

[85] Zhao D.Y., Ni Z., Pu S., Zhong G., Schmitges F.W., Braunschweig U. (2019). Regulation of transcription termination by FUS and TDP-43. bioRxiv.

[86] Gianini M., Bayona-Feliu A., Sproviero D., Barroso S.I., Cereda C., Aguilera A. (2020). TDP-43 mutations link amyotrophic lateral sclerosis with R-loop homeostasis and R loopmediated DNA damage. PLoS Genet..

[87] Nie X., Xu Q., Xu C., Chen F., Wang Q., Qin D. (2023). Maternal TDP-43 interacts with RNA Pol II and regulates zygotic genome activation. Nat Commun.

[88] Morera A.A., Ahmed N.S., Schwartz J.C. (2019). TDP-43 regulates transcription at protein-coding genes and alu retrotransposons. Biochim. Biophys. Acta. Gene Regul Mech..

[89] Hou Y., Li Y., Xiang J.F., Tilahun K., Jiang J., Corces V.G. (2024). TDP-43 chronic deficiency leads to dysregulation of transposable elements and gene expression by affecting R-loop and 5hmC crosstalk. Cell Rep..

[90] Kapeli K., Pratt G.A., Vu A.Q., Hutt K.R., Martinez F.J., Sundararaman B. (2016). Distinct and shared functions of ALS-associated proteins TDP-43, FUS and TAF15 revealed by multisystem analyses. Nat. Commun..

[91] Thompson V.F., Wieland D.R., Mendoza-Leon V., Janis H.I., Lay M.A., Harrell L.M. (2023). Binding of the nuclear ribonucleoprotein family member FUS to RNA prevents R-loop RNA:DNA hybrid structures. J. Biol. Chem..

[92] Fang M., Deibler S.K., Nana A.L., Vatsavayai S.C., Banday S., Zhou Y. (2023). Loss of TDP-43 function contributes to genomic instability in amyotrophic lateral sclerosis. Front. Neurosci..

[93] Eréndira Avendaño-Vázquez S., Dhir A., Bembich S., Buratti E., Proudfoot N., Baralle F.E. (2012). Autoregulation of TDP-43 mRNA levels involves interplay between transcription, splicing, and alternative polyA site selection. Genes Dev..

[94] Polymenidou M., Lagier-Tourenne C., Hutt K.R., Huelga S.C., Moran J., Liang T.Y. (2011). Long pre-mRNA depletion and RNA missplicing contribute to neuronal vulnerability from loss of TDP-43. Nat. Neurosci..

[95] Rot G., Wang Z., Huppertz I., Modic M., Lenče T., Hallegger M. (2017). High-resolution RNA maps suggest common principles of splicing and polyadenylation regulation by TDP-43. Cell Rep..

[96] Wang W.Y., Pan L., Su S.C., Quinn E.J., Sasaki M., Jimenez J.C. (2013). Interaction of FUS and HDAC1 regulates DNA damage response and repair in neurons. Nat. Neurosci..

[97] Mitra J., Guerrero E.N., Hegde P.M., Liachko N.F., Wang H., Vasquez V. (2019). Motor neuron disease-associated loss of nuclear TDP-43 is linked to DNA double-strand break repair defects. Proc. Natl. Acad. Sci. U. S. A..

[98] Hicks G.G., Singh N., Nashabi A., Mai S., Bozek G., Klewes L. (2000). Fus deficiency in mice results in defective B-lymphocyte development and activation, high levels of chromosomal instability and perinatal death. Nat. Genet..

[99] Kuroda M., Sok J., Webb L., Baechtold H., Urano F., Yin Y. (2000). Male sterility and enhanced radiation sensitivity in TLS(-/-) mice. EMBO J..

[100] Ginno P.A., Lim Y.W., Lott P.L., Korf I., Chédin F. (2013). GC skew at the 59 and 39 ends of human genes links R-loop formation to epigenetic regulation and transcription termination. Genome Res..

[101] Groh M., Lufino M.M.P., Wade-Martins R., Gromak N. (2014). R-loops associated with triplet repeat expansions promote gene silencing in Friedreich ataxia and fragile X syndrome. PLoS Genet..

[102] Colak D., Zaninovic N., Cohen M.S., Rosenwaks Z., Yang W.Y., Gerhardt J. (2014). Promoter-bound trinucleotide repeat mRNA drives epigenetic silencing in fragile X syndrome. Science (1979).

[103] Butler J.S., Napierala M. (2015). Friedreich’s ataxia – a case of aberrant transcription termination?. Transcription.

[104] Haeusler A.R., Donnelly C.J., Periz G., Simko E.A.J., Shaw P.G., Kim M.S. (2014). C9orf72 nucleotide repeat structures initiate molecular cascades of disease. Nature.

[105] Chédin F. (2016). Nascent connections: R-Loops and chromatin patterning. Trends Genet..

[106] El Hage A., French S.L., Beyer A.L., Tollervey D. (2010). Loss of Topoisomerase I leads to R-loop-mediated transcriptional blocks during ribosomal RNA synthesis. Genes Dev..

[107] Yasuhara T., Kato R., Hagiwara Y., Shiotani B., Yamauchi M., Nakada S. (2018). Human Rad52 promotes XPG-mediated R-loop processing to initiate transcription-associated homologous recombination repair. Cell.

[108] Liang Z., Liang F., Teng Y., Chen X., Liu J., Longerich S. (2019). Binding of FANCI-FANCD2 complex to RNA and R-Loops stimulates robust FANCD2 monoubiquitination. Cell Rep..

[109] García-Rubio M.L., Pérez-Calero C., Barroso S.I., Tumini E., Herrera-Moyano E., Rosado I.V. (2015). The Fanconi Anemia pathway protects genome integrity from R-loops. PLoS Genet..

[110] Skourti-Stathaki K., Proudfoot N.J. (2014). A double-edged sword: r loops as threats to genome integrity and powerful regulators of gene expression. Genes Dev..

[111] Barmada S.J. (2015). Linking RNA dysfunction and neurodegeneration in amyotrophic lateral sclerosis. Neurotherapeutics.

[112] Butti Z., Patten S.A. (2019). RNA dysregulation in amyotrophic lateral sclerosis. Front. Genet..

[113] Zylka M.J., Simon J.M., Philpot B.D. (2015). Gene length matters in neurons. Neuron.

[114] Perego M.G.L., Taiana M., Bresolin N., Comi G.P., Corti S. (2019). R-Loops in motor neuron diseases. Mol Neurobiol.

[115] Kim B.W., Jeong Y.E., Wong M., Martin L.J. (2020). DNA damage accumulates and responses are engaged in human ALS brain and spinal motor neurons and DNA repair is activatable in iPSC-derived motor neurons with SOD1 mutations. Acta Neuropathol. Commun..

[116] Nitoiu A., Nabeel-Shah S., Farhangmehr S., Pu S., Braunschweig U., Blencowe B.J. (2021). KRAB Zinc Finger protein Znf684 interacts with Nxf1 to regulate mRNA export. bioRxiv.

[117] Mak A.B., Ni Z., Hewel J.A., Chen G.I., Zhong G., Karamboulas K. (2010). A lentiviral functional proteomics approach identifies chromatin remodeling complexes important for the induction of pluripotency. Mol. Cell Proteomics.

[118] Nabeel-Shah S., Lee H., Ahmed N., Burke G.L., Farhangmehr S., Ashraf K. (2022). SARS-CoV-2 nucleocapsid protein binds host mRNAs and attenuates stress granules to impair host stress response. iScience.

[119] Boguslawski S.J., Smith D.E., Michalak M.A., Mickelson K.E., Yehle C.O., Patterson W.L. (1986). Characterization of monoclonal antibody to DNA · RNA and its application to immunodetection of hybrids. J Immunol. Methods..

[120] Song J., Nabeel-Shah S., Pu S., Lee H., Braunschweig U., Ni Z. (2022). Regulation of alternative polyadenylation by the C2H2-zinc-finger protein Sp1. Mol. Cell.

[121] Langmead B., Salzberg S.L. (2012). Fast gapped-read alignment with Bowtie 2. Nat. Methods.

[122] Kharchenko P.V., Tolstorukov M.Y., Park P.J. (2008). Design and analysis of ChIP-seq experiments for DNA-binding proteins. Nat. Biotechnol..

[123] Ramírez F., Dündar F., Diehl S., Grüning B.A., Manke T. (2014). deepTools: a flexible platform for exploring deep-sequencing data. Nucleic Acids Res.

[124] Bailey T.L. (2011). DREME: motif discovery in transcription factor ChIP-seq data. Bioinformatics.

[125] Nabeel-Shah S., Pu S., Burke G.L., Ahmed N., Braunschweig U., Farhangmehr S. (2024). Recruitment of the m6A/m6Am demethylase FTO to target RNAs by the telomeric zinc finger protein ZBTB48. Genome Biol..

[126] Nabeel-Shah S., Greenblatt J. (2023). Revised iCLIP-seq protocol for profiling RNA-protein interaction sites at individual nucleotide resolution in living cells. Bio Protoc.

[127] Bolger A.M., Lohse M., Usadel B. (2014). Trimmomatic: a flexible trimmer for Illumina sequence data. Bioinformatics.

[128] Trapnell C., Pachter L., Salzberg S.L. (2009). TopHat: discovering splice junctions with RNA-Seq. Bioinformatics.

