# Supplementary material for: RNA-binding proteins TDP-43 and FUS promote R-loop resolution and regulate transcription termination
Source: J Biol Chem. 2026 Mar 6;302(5):111348. doi: 10.1016/j.jbc.2026.111348 (PMC13068857; doi:10.1016/j.jbc.2026.111348)
Supplement: Supplementary Material 1 [file mmc1.pdf]

Supplementary Figure S1

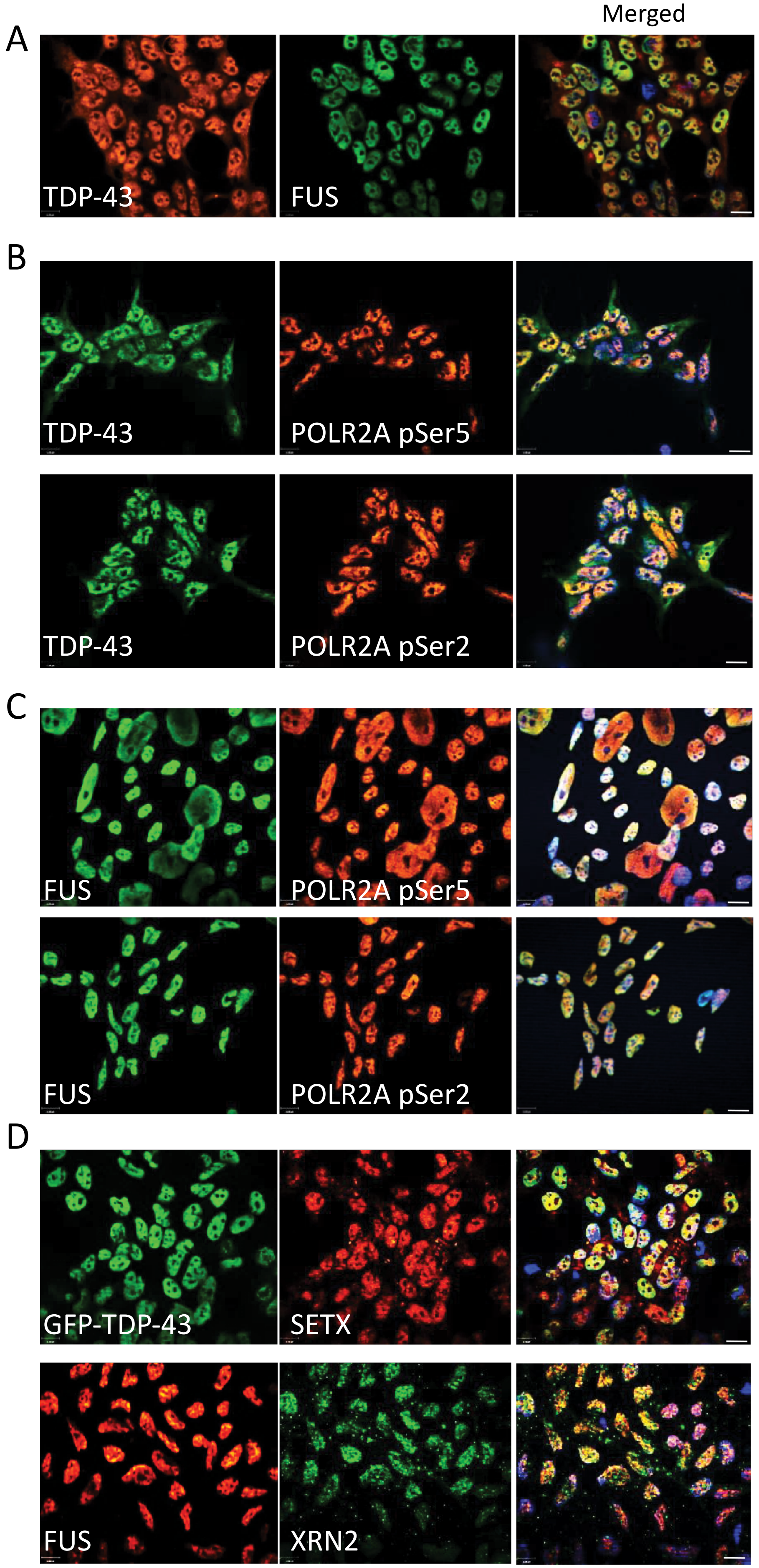

**Supplementary Figure S1: FUS and TDP-43 localize in the nuclei.**

**A.** Immunostaining of FUS and TDP-43 in HEK293 cells. **B-D**: The staining indicates that FUS, TDP-43, SETX, XRN2, and POLR2A (pSer2, pSer5) all show nuclear localization, as expected. Hoechst stain for DNA shown in blue. Note that GFP-TDP43 (panel D) also shows nuclear staining, similar to the endogenous TDP-43. Scale bars: 10 μm.

# Supplementary Figure S2

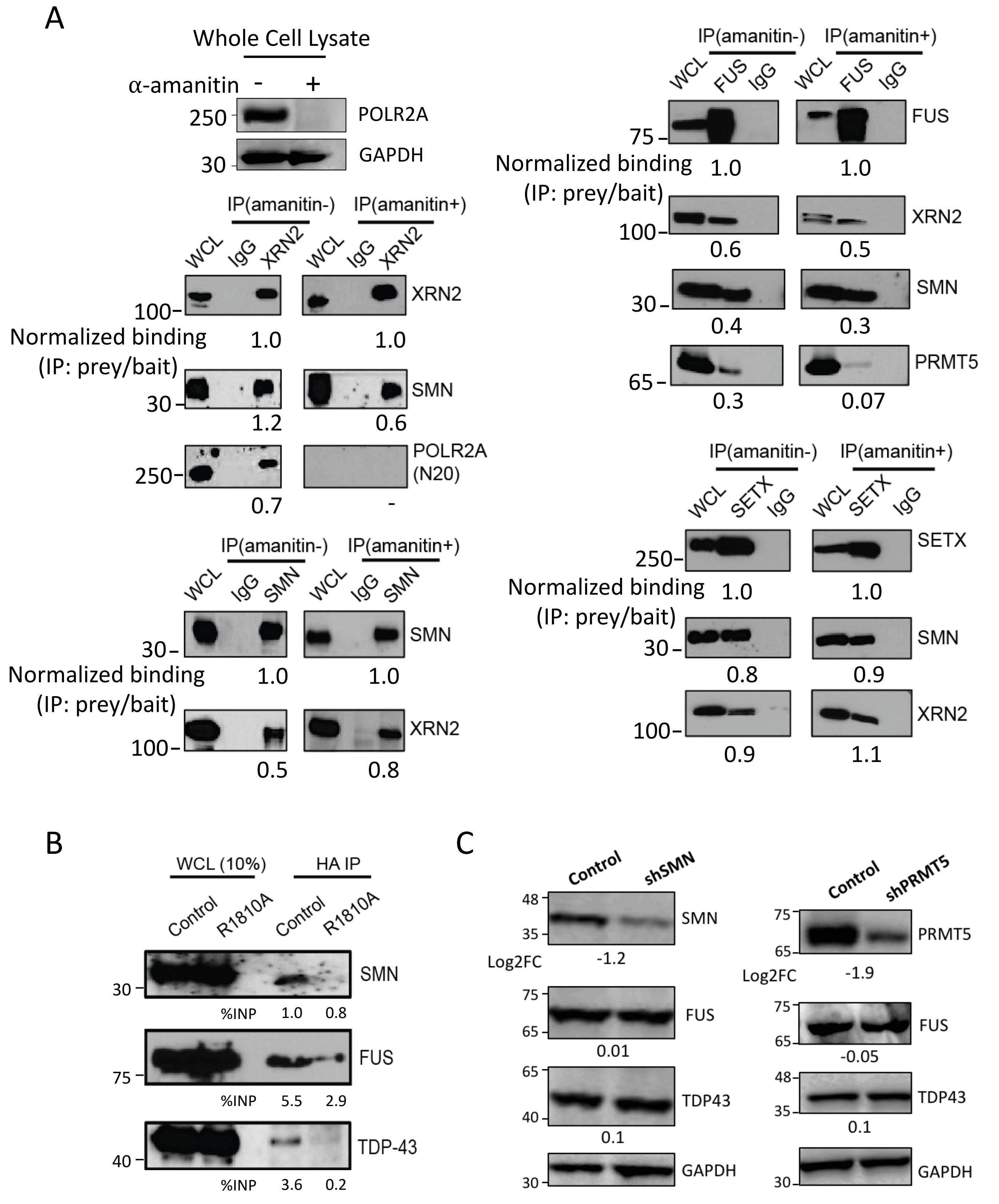

**Supplementary Figure S2: SMN and FUS interact with termination factors independently of RNAPII, and the POLR2A R1810A mutation disrupts the interactions amongst RNAPII, SMN, TDP-43, and FUS.**

**A.** Top left, Western blotting analysis using whole cell lysates (WCLs) prepared from either α-amanitin treated or untreated HEK293 cells. The blot was probed with the indicated antibodies. All remaining blots: IP with the indicated antibodies from HEK293 WCLs, followed by western blotting with the indicated antibodies with or without α-amanitin pre-treatment of the HEK293 cells to degrade POLR2A. Many of the interactions persist independently of RNAPII, including SMN-XRN2, SMN-FUS, SETX-XRN2, SETX-FUS, and FUS-XRN2. Bands were quantified, and normalized binding (IP: prey/bait) values are shown below each IP band. The experiments were repeated in at least two biological replicates. **B.** IP with the indicated antibodies from WCLs from Raji cells stably expressing the HA-tagged wild-type or R1810A mutant POLR2A after 3-days of α-amanitin treatment (2 ug/mL) that abolishes the endogenous POLR2A. Anti-HA was used to precipitate HA-tagged wild-type (Control) or R1810A mutant POLR2A, followed by western blotting with the indicated antibodies. Bands were quantified and %INP values are shown below each IP band. **C:** Western blotting analysis using WCLs prepared from either shRNA-treated or control cells. Blots were probed with the indicated antibodies. Log2-fold-change (Log2FC) was calculated using the adjusted signal intensities of the bands in shRNA-treated and control lanes (adj_KD/adj_ Control), where the adjusted (adj) intensities represent GAPDH normalized values for each band. Signal intensities were calculated using ImageJ software (*n* = *2*).

Supplementary Figure S3

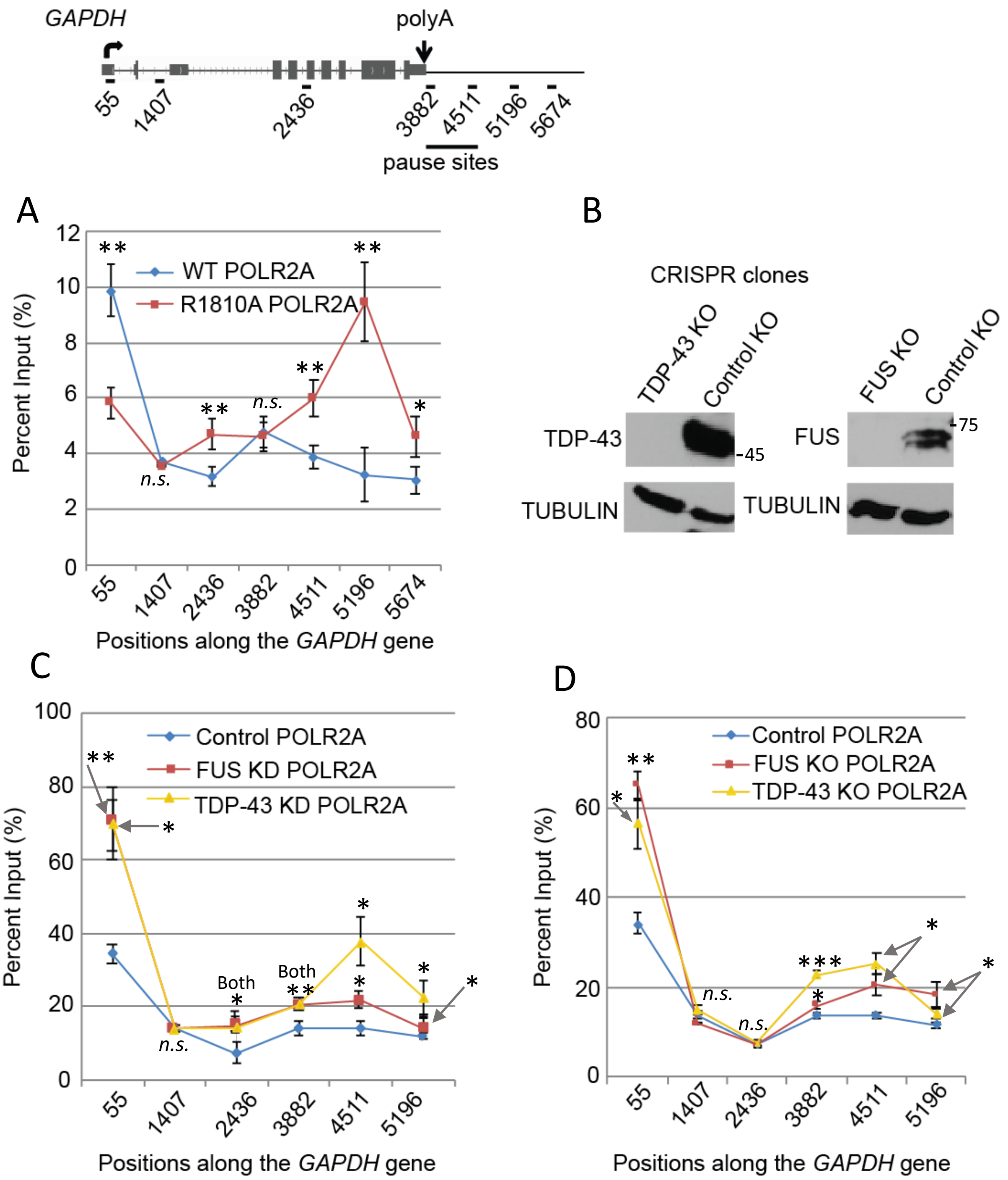

**Supplementary Figure S3: RNAPII R1810, FUS and TDP-43 regulate RNAPII termination on the *GAPDH* gene.**

**A.** ChIP quantification of WT and the endogenous R1810A mutant RNAPII using POLR2A antibody (8WG16, N20) in HEK293 cells, using the indicated primer positions for qPCR along the *GAPDH* gene. Error bars denote standard error of mean (s.e.m.) of biological replicates ($n$ = 4). **B.** Western blotting with the indicated antibodies verifies the knock-out of TDP-43 or FUS by the CRISPR/Cas9 system. **C.** Quantification of RNAPII ChIP using POLR2A antibody (4H8, N20) in HEK293 cells, using the indicated primer positions for qPCR along the *GAPDH* gene, after stably knocking down FUS or TDP-43, with GFP knock-down as a negative control. Error bars denote s.e.m. biological replicates ($n$ = 3). **D.** Quantification of RNAPII ChIP using POLR2A antibody (4H8, N20) in HEK293 cells, using the indicated primer positions for qPCR along the *GAPDH* gene, after knocking out FUS or TDP-43 using the CRISPR/Cas9 system or using scrambled guide RNA as negative control. Error bars denote s.e.m. of biological replicates ($n$ = 3). ChIP signals are normalized to the gene body region (1407 or 2436) across samples and replicates.

Note: P-values were calculated using two-tailed Student's t-test for the indicated sites, $***p \leq 0.001$, $**p \leq 0.01$, $*p \leq 0.05$, n.s.: non-significant.

Supplementary Figure S4

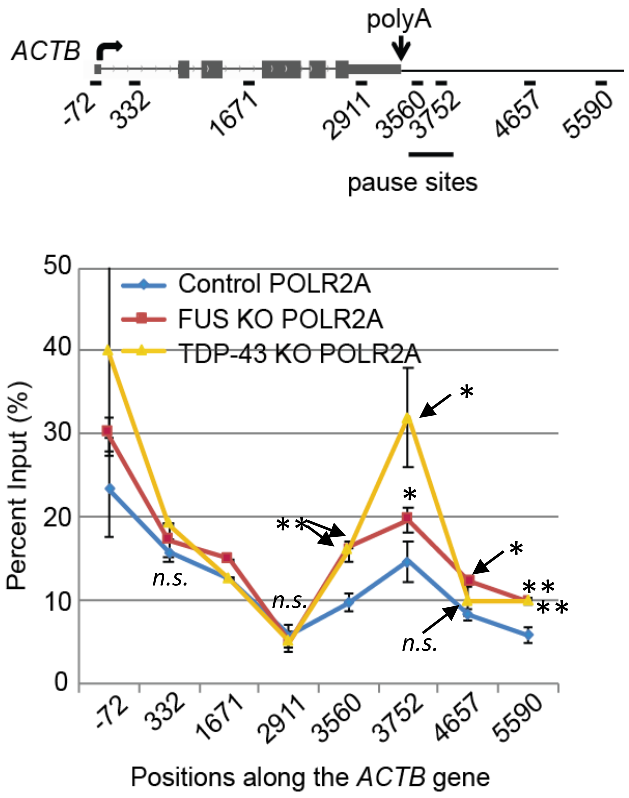

**Supplementary Figure S4: FUS and TDP-43 regulate RNAPII termination on the *ACTB* gene.**

Quantification of RNAPII ChIP using POLR2A antibody (4H8, N20) in HEK293 cells, using the indicated primer positions for qPCR along the *ACTB* gene after knocking out FUS or TDP-43 with the CRISPR/Cas9 system, or using scrambled guide RNA as negative control. Error bars denote s.e.m. of biological replicates ($n$ = 5). Note: P-values were calculated using two-tailed Student's t-test for the indicated sites, $***p \leq 0.001$, $**p \leq 0.01$, $*p \leq 0.05$, n.s.: non-significant.

Supplementary Figure S5

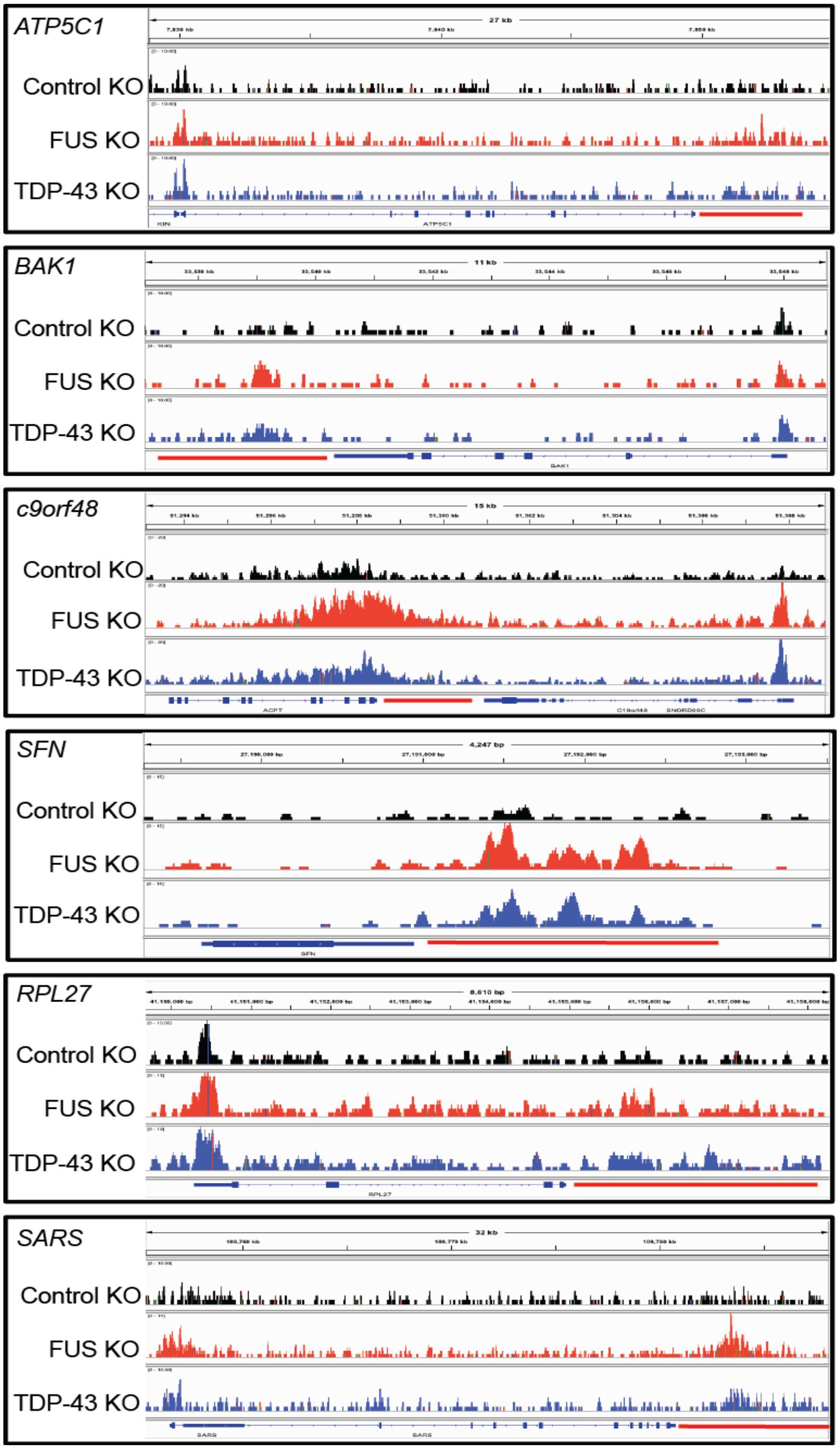

**Supplementary Figure S5: FUS and TDP-43 regulate RNAPII promoter and terminator stalling.**

RNAPII ChIP-seq results for several housekeeping genes are displayed in detail with the Integrative Genomics Viewer. RNAPII termination regions are underlined in red.

Supplementary Figure S6

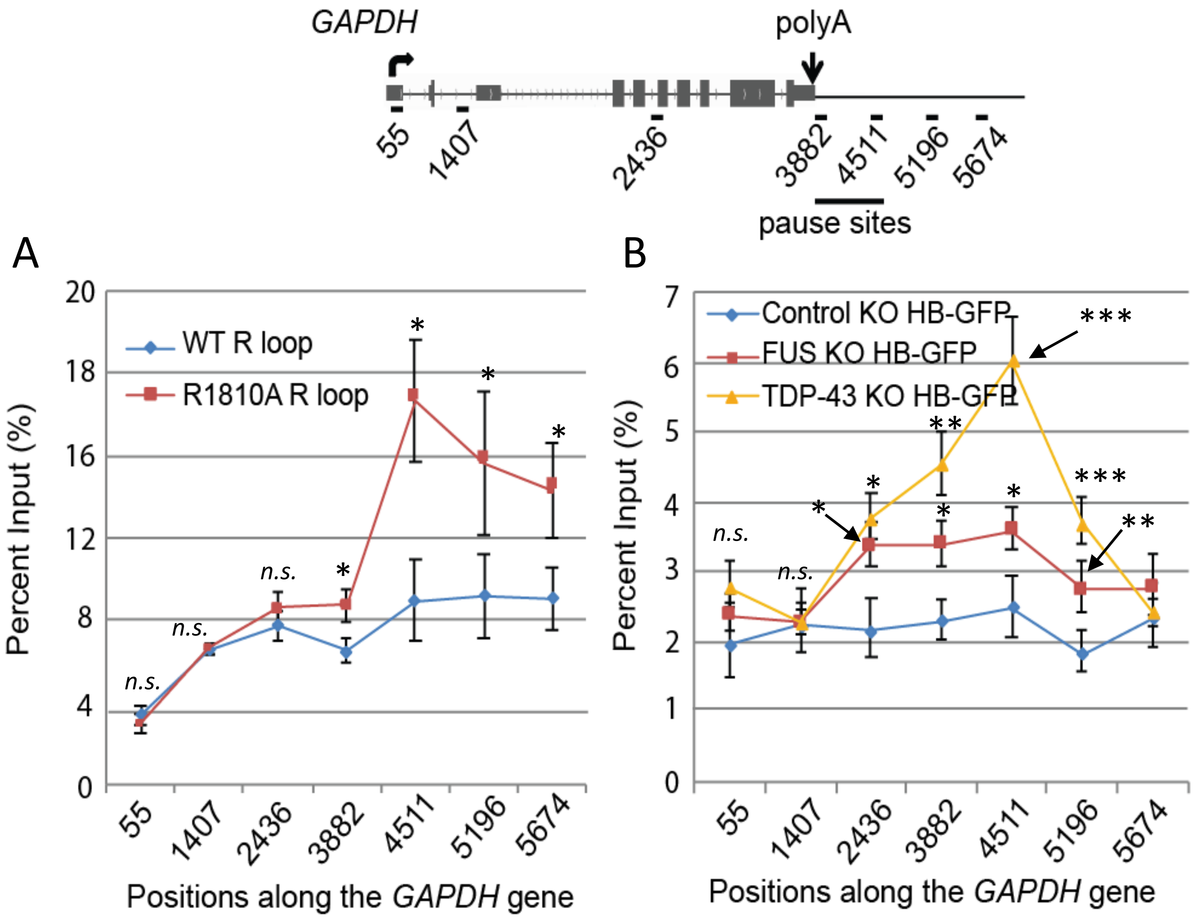

**Supplementary Figure S6: RNAPII R1810, FUS, and TDP-43 are important for resolving R-loops in RNAPII termination regions.**

**A.** Quantification of R-loops with the S9.6 antibody for the WT and the endogenous R1810A mutant RNAPII in HEK293 cells, with the indicated primer positions for qPCR along the *GAPDH* gene. Error bars denote s.e.m. of biological replicates ($n$ =3).

**B.** Quantification of R-loops with the GFP-HB construct, with the indicated primer positions for qPCR along the *GAPDH* gene, after knocking out FUS or TDP-43 with the CRISPR/Cas9 system or with the scrambled guide RNA as a negative control. Error bars denote s.e.m. of biological replicates ($n$ = 3). R-loop signals are normalized to the gene body region (1407) across samples and replicates.

Note: All P-values were calculated using two-tailed Student's t-test for the indicated sites, ∗∗∗$p \leq 0.001$, ∗∗$p \leq 0.01$, ∗$p \leq 0.05$, n.s.: non-significant.

# Supplementary Figure S7

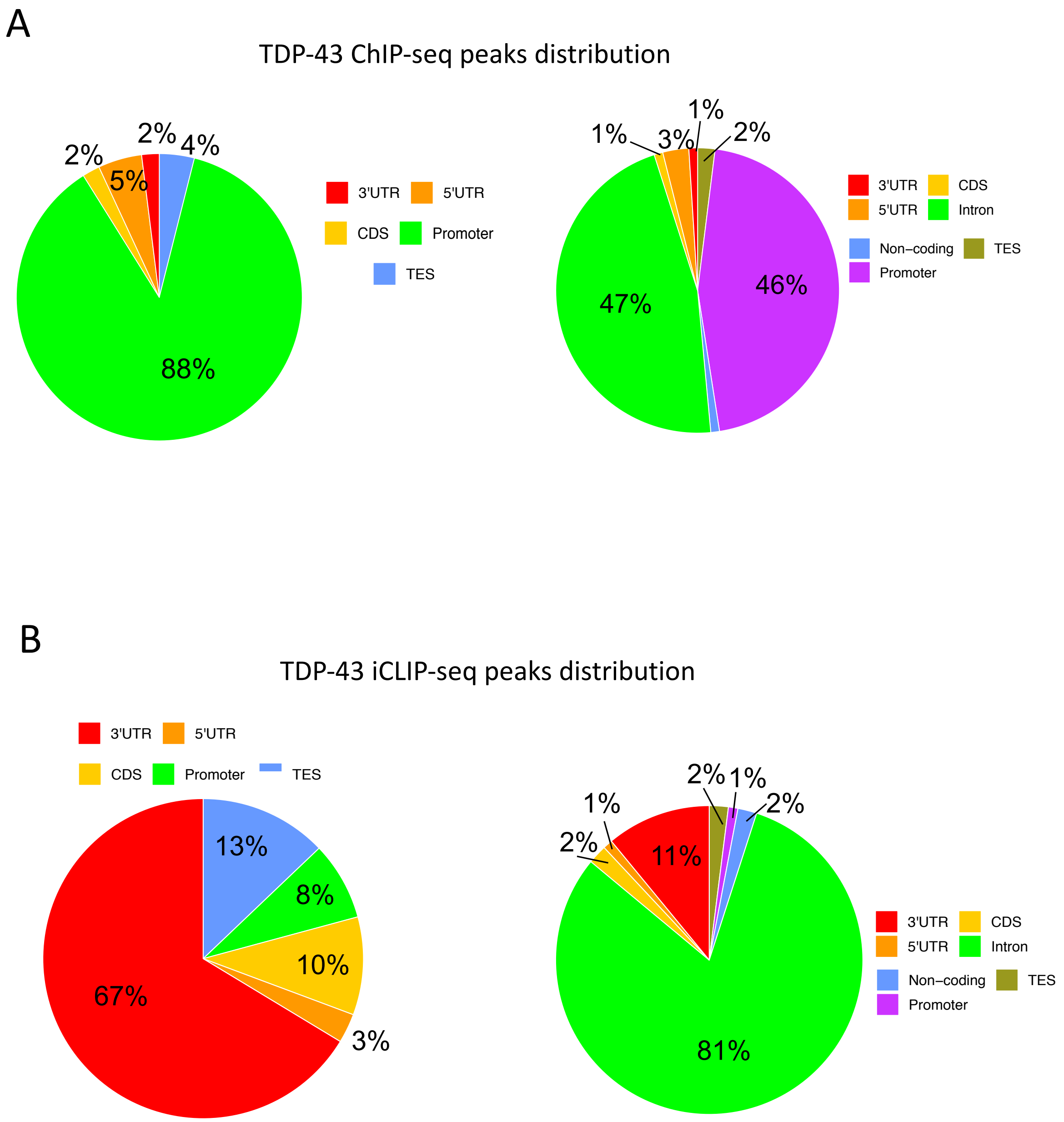

**Supplementary Figure S7: TDP-43 DNA- and RNA-binding preferences A:** Pie chart representation of TDP-43 ChIP-seq peaks distribution across the indicated regions (left: Peak distribution without including the introns in the genomic features). **B:** Pie chart representation of TDP-43 iCLIP-seq peaks distribution across the indicated regions (left: Peak distribution without including the introns in the genomic features).

# Supplementary Figure S8

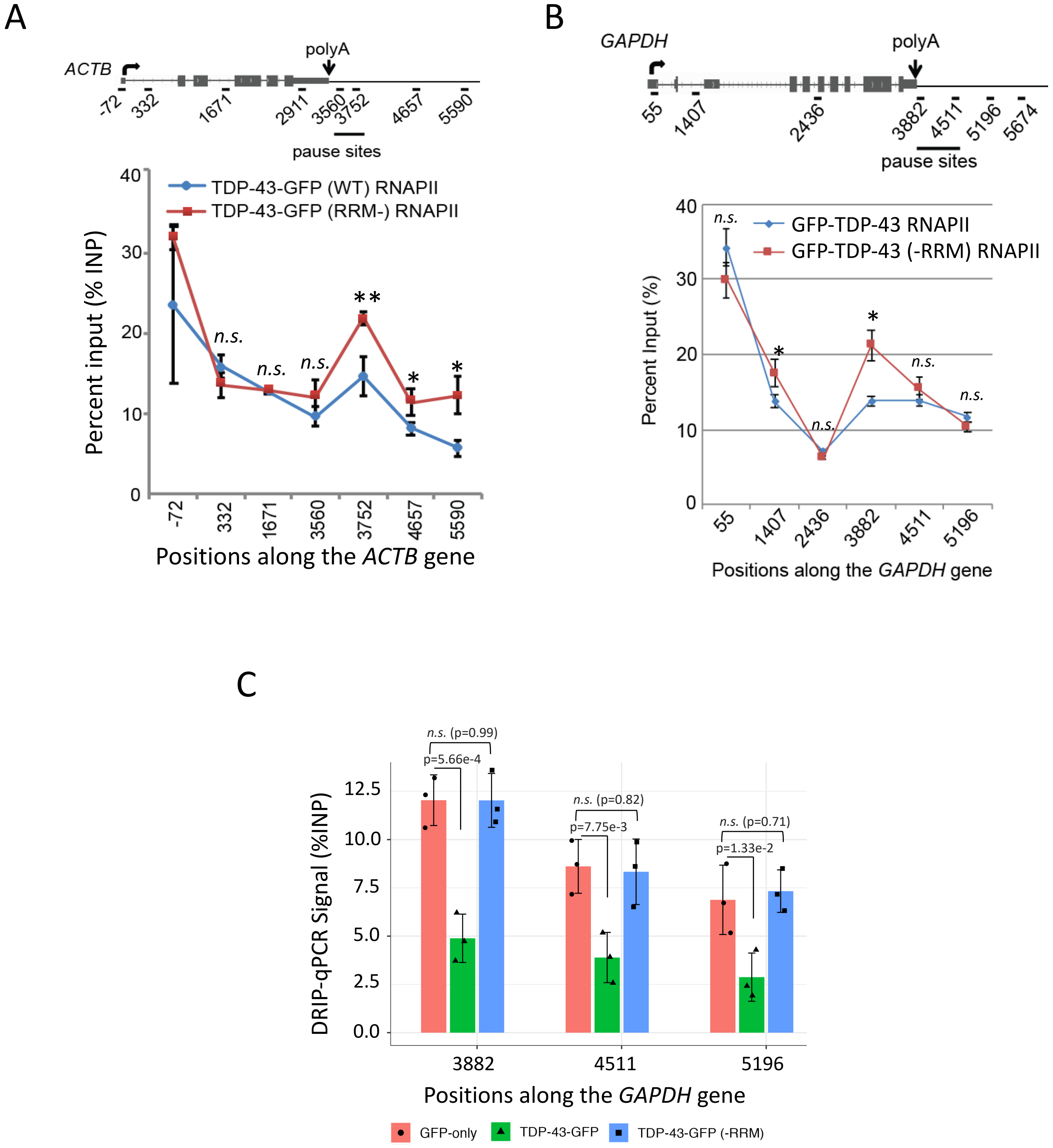

**Supplementary Figure S8: RNA recognition motifs (RRM) of TDP-43 are important for TDP-43 to regulate RNAPII termination on the *GAPDH* gene**

**A.** Quantification of RNAPII ChIP using the POLR2A antibody (N20) in HEK293 cells, with cells overexpressing wild type GFP-TDP-43 or the truncation mutant lacking RNA binding domains GFP-TDP-43 (RRM-), using the indicated primer positions for qPCR along the *ACTB* gene. Error bars denote s.e.m. of biological replicates ($n$ = $3$). P-values were calculated using two-tailed Student's t-test for the indicated sites, $***p \leq 0.001$, $**p \leq 0.01$, $*p \leq 0.05$, n.s.: non-significant. ChIP signals are normalized to the gene body region (1671) across samples and replicates. **B.** Quantification of RNAPII ChIP using the POLR2A antibody (N20) with cells overexpressing wild type GFP-TDP-43 or the GFP-TDP-43 (RRM-) truncation mutant, using the indicated primer positions for qPCR along the *GAPDH* gene. Error bars denote s.e.m. of biological replicates ($n$ = $3$). P-values were calculated using two-tailed Student's t-test for the indicated sites, $***p \leq 0.001$, $**p \leq 0.01$, $*p \leq 0.05$, n.s.: non-significant. R-loop signals are normalized to the gene body region (2436) across samples and replicates. **C.** Quantification of R-loops in the *GAPDH* gene. Error bars denote standard deviation (s.d.) of biological replicates ($n$ = $3$). P-values were calculated using two-tailed Student's t-test, p-values are indicated, n.s.: non-significant.

Supplementary Figure S9

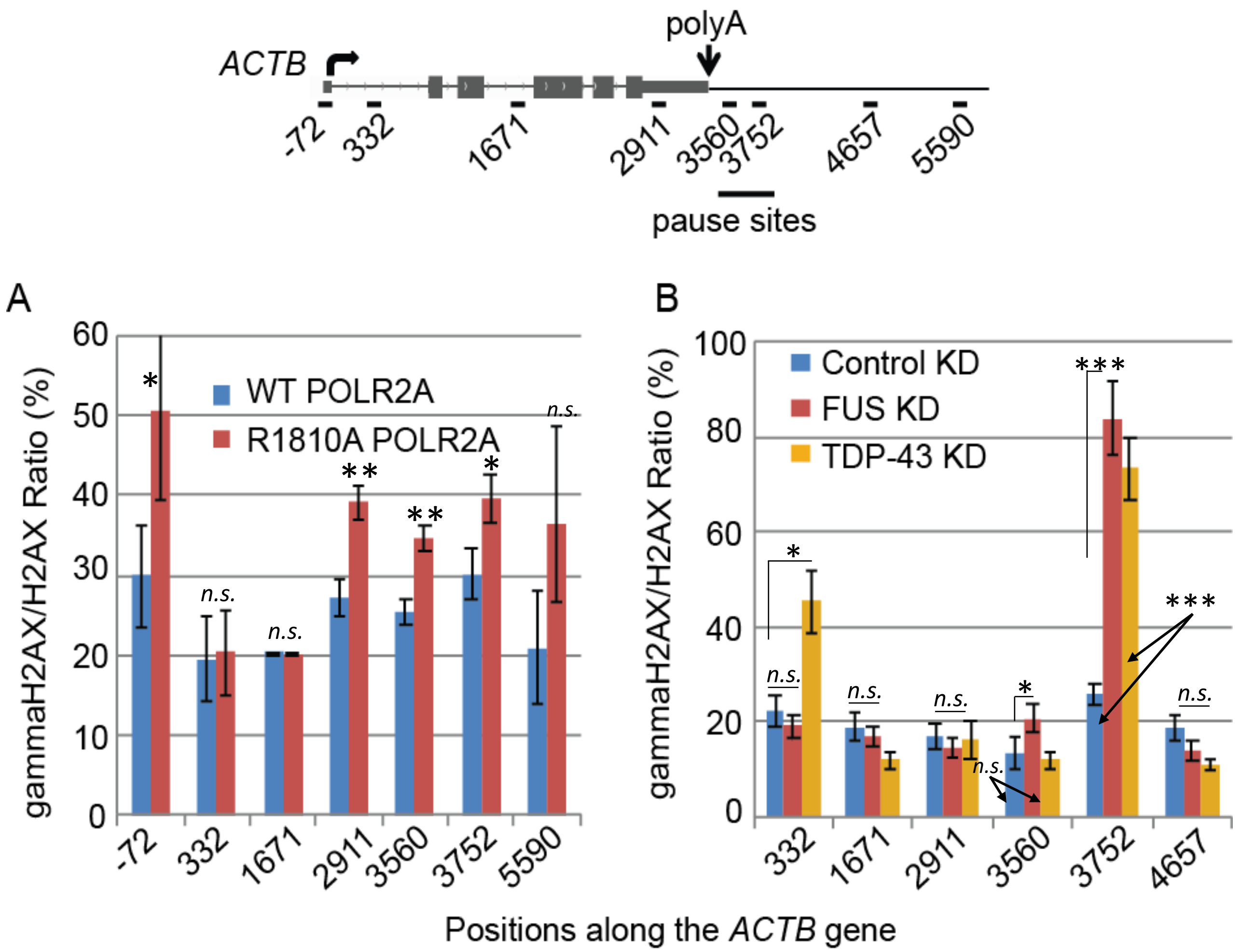

**Supplementary Figure S9: RNAPII CTD R1810, FUS and TDP-43 prevent DNA damage at RNAPII transcription terminators.**

**A, B.** ChIP quantifications of the γH2AX/H2AX ratio in HEK293 cells along the length of the *ACTB* gene, comparing WT and the endogenous R1810A mutant POLR2A, normalized to the gene body region (1671) (**A**), or after knocking down FUS or TDP-43, with the knock-down of GFP as a negative control (**B**). Error bars denote s.e.m. of biological replicates ($n$ = 4). Note: P-values were calculated using two-tailed Student's t-test for the indicated sites, $***p \leq 0.001$, $**p \leq 0.01$, $*p \leq 0.05$, n.s.: non-significant.
