# Supplementary material for: RNA-binding proteins TDP-43 and FUS promote R-loop resolution and regulate transcription termination
Source: J Biol Chem. 2026 Mar 6;302(5):111348. doi: 10.1016/j.jbc.2026.111348 (PMC13068857; doi:10.1016/j.jbc.2026.111348)
Supplement: Supplementary Material 2 [file mmc2.pdf]

Supplementary Table 1: *RNAPII 3'-end processing, elongation, and termination factors that contain arginine dimethylations as potential SMN binders*

**RNAPII 3'-end processing and termination factors**	**Detection method**
CPSF5	Rme2s Antibody IP-MS
CPSF6 (GR)	Rme2s Antibody IP-MS
CPSF1	Rme2s Antibody IP-MS
CSTF2 (PRG, TRG)	SILAC labeling for MS
PABP1 (NR, LR, PR)	Rme2s Antibody IP-MS
PABP2 (PR, GR)	Rme2s Antibody IP-MS
PABP4 (PR)	SILAC labeling for MS
DHX9 (PRP, QRG, GRG)	SILAC labeling for MS
PCF11 (PR)	SILAC labeling for MS
WDR33 (GR)	SILAC labeling for MS
EF1a1/2	SILAC labeling for MS
DDX20	SILAC labeling for MS
RBM9	SILAC labeling for MS
RBBP6	Rme Antibody IP-MS
TAF15 (GR)	SILAC labeling for MS
G3BP1/2 (GR, PR)	SILAC labeling for MS
FCP1 (GR, PR)	Rme2s Antibody IP-MS
SPT5 (GR)	Rme2s Antibody IP-MS
EWSR1 (GR)	Rme2s Antibody IP-MS
FUS (GR)	Rme2s Antibody IP-MS
TDP-43 (GR)	Rme Antibody IP-MS<br>SILAC labeling for MS
XRN2 (GR, PRG, YRP)	SILAC labeling for MS
SETX	SILAC labeling for MS

Supplementary Table 2: *Genes implicated in the pathology of ALS that often lead to the formation of cytoplasmic inclusion bodies that trap TDP-43 or FUS.*

**ALS Mutated Genes**	**Frequency**	**Proteins trapped in cytoplasmic inclusion bodies**	
		TDP-43	FUS
C9ORF72 expansion	40%	yes	
Superoxide Dismutase (SOD1)	20%		
Granulin(GRN)	low	yes	
Angiogenin, Ribonuclease (ANG)	low	yes	
Ubiquilin 2 (UBQLN2)	low	yes	
Profilin 1 (PFN1)	low	yes	
valosin containing protein (VCP)	low	yes	
hnRNPA1/B1	low	yes	
Sequestosome 1 (SQSTM1)	low	yes	
Ataxin 2 (ATXN2)	low	yes	
Optineurin (OPTN)	low	yes	
TAR DNA binding protein (TDP-43)	low	yes	
Fused in Sarcoma (FUS)	low		yes
TATA-Binding Protein-Associated Factor (TAF15)	low		Yes
EWS RNA-binding protein 1 (EWSR1)	low		yes
Senataxin (SETX)	low		
